# Projecting results of zoned multi-environment trials to new locations using environmental covariates with random coefficient models: accuracy and precision

**DOI:** 10.1007/s00122-021-03786-2

**Published:** 2021-04-08

**Authors:** Harimurti Buntaran, Johannes Forkman, Hans-Peter Piepho

**Affiliations:** 1 Biostatistics Unit, Institute of Crop Science, University of Hohenheim, Fruwirthstraße 23, 70599 Stuttgart, Germany; 2 Department of Crop Production Ecology, Swedish University of Agricultural Sciences, Box 7043, 750 07 Uppsala, Sweden

## Abstract

**Key message:**

We propose the utilisation of environmental covariates in random coefficient models to predict the genotype performances in new locations.

**Abstract:**

Multi-environment trials (MET) are conducted to assess the performance of a set of genotypes in a target population of environments. From a grower’s perspective, MET results must provide high accuracy and precision for predictions of genotype performance in new locations, i.e. the grower’s locations, which hardly ever coincide with the locations at which the trials were conducted. Linear mixed modelling can provide predictions for new locations. Moreover, the precision of the predictions is of primary concern and should be assessed. Besides, the precision can be improved when auxiliary information is available to characterize the targeted locations. Thus, in this study, we demonstrate the benefit of using environmental information (covariates) for predicting genotype performance in some new locations for Swedish winter wheat official trials. Swedish MET locations can be stratified into zones, allowing borrowing information between zones when best linear unbiased prediction (BLUP) is used. To account for correlations between zones, as well as for intercepts and slopes for the regression on covariates, we fitted random coefficient (RC) models. The results showed that the RC model with appropriate covariate scaling and model for covariate terms improved the precision of predictions of genotypic performance for new locations. The prediction accuracy of the RC model was competitive compared to the model without covariates. The RC model reduced the standard errors of predictions for individual genotypes and standard errors of predictions of genotype differences in new locations by 30–38% and 12–40%, respectively.

**Supplementary information:**

The online version contains supplementary material available at (10.1007/s00122-021-03786-2).

## Introduction

The main goal of a plant breeding programme is to develop well-adapted genotypes in a target population of environments (TPE). Multi-environment trials (MET) are conducted to evaluate candidate genotypes in the TPE, and to understand and exploit the pattern of genotype × environment interactions (GEI) in the TPE. GEI is the differential response of genotypes across different environments (Kang and Gorman 1989). GEI in a TPE can be exploited to make more targeted predictions and recommendations on cultivars. This is of particular interest when there is crossover interaction, which poses a challenge when selecting genotypes for broad adaptation.

Identification of environmental covariates that are responsible for GEI is useful to enhance the predictive capability of MET analyses (Heslot et al. 2014) and evaluate the adaptability of the genotypes to the new target environment. The most commonly used types of environmental covariates are soil and meteorological covariates (van Eeuwijk et al. 2016). Incorporating environmental covariates in the GEI analysis has been done by factorial regression (Denis 1988; Piepho et al. 1998; van Eeuwijk and Elgersma 1993). Furthermore, environmental covariates have been used in a linear mixed model framework, such as in quantitative trait loci (QTL) biparental mapping to dissect the response of marker effects to the environmental covariates known as QTL × environment interactions (Boer et al. 2007; Crossa et al. 1999; Malosetti et al. 2004) or as eco-physiological QTL (van Eeuwijk et al. 2005). Environmental covariates have also been introduced in genomic selection (Gillberg et al. 2019; Heslot et al. 2014; van Eeuwijk et al. 2019).

The regression on environmental covariates is usually modelled by fixed effects. This type of modelling is appropriate when only studying the pattern of GEI at the tested locations. Such models are also appropriate for making predictions in an unstructured TPE. However, when the TPE is sub-divided into zones, it is necessary to model genotypic effects as random in order to borrow strength between zones (Buntaran et al. 2019, 2020). If such modelling is coupled with factorial regression approaches, genotype-specific regression coefficients must be modelled as random effects as well, giving rise to what are known as random coefficient (RC) models (Longford 1993; Milliken and Johnson 2002).

Although MET are usually designed to cover the whole TPE, none of the trials in an MET coincides exactly with a grower’s field or location. Thus, grower’s fields, the real target of breeding, must be seen as new locations in the TPE. In the same vein, it may be said that the MET analysis is usually used to produce predictions of tested genotypes for a new location, making use of the information from tested locations. Predicting genotype performance in a new location is akin to predicting values that have no records at all. Henderson (1977) showed that best linear unbiased prediction (BLUP) can be used to predict breeding values for the animals that had no records. BLUP, therefore, can also be used to obtain genotype predictions in a new location that has no records.

Since growers’ fields hardly ever coincide with the trials’ location and, in practice, cultivar yield will never reach the exact same value as the predicted mean values from the MET, reporting the precision or precision measures as quantified by standard errors and prediction intervals is highly desirable. Without this, growers are left with having no information regarding the precision in the predictions that are reported. The key challenge is that the standard errors of predictions of variety means obtained from the routine analysis of MET are only valid for the locations where the trials were carried out, but not for the untested locations or growers’ field. However, the precision of the predictions for the untested locations is crucial in order to assist growers in selecting a cultivar for their farm or field.

Accuracy refers to how close the value of the statistic is to a supposed ‘‘true value’’. In other words, accuracy measures how close an estimate $\widehat{\theta }$ of a parameter $\theta$ is to the ‘‘true value’’ of $\theta$ (Kotz et al. 2006). Accuracy can be evaluated via a cross-validation (CV) study by estimating prediction error (Hastie et al. 2009). From a CV study, accuracy can be measured in terms of mean squared error (MSE), which consists of variance and squared bias of $\widehat{\theta }$. Conversely, the precision of an estimator $\widehat{\theta }$, measures how narrow the distribution of $\widehat{\theta }$ clusters about its expected value (Kotz et al. 2006). The precision of $\widehat{\theta }$ is the reciprocal of the variance of $\widehat{\theta }$.

In this study, we explore the use of the RC models for improving the precision of yield predictions in some new locations, which represent growers’ fields, and evaluate the prediction accuracy. The precision of predictions is assessed with standard errors of predictions of genotypic values (SEPV) and standard errors of the predictions of pairwise differences of genotypic values (SEPD). The prediction accuracy was evaluated via a CV study.

## Materials and methods

### Dataset

In this study, a dataset from Swedish official cultivar testing in 2016 was used. The dataset comprises 25 genotypes of winter wheat tested in 18 locations. In addition, the dataset includes information about four new locations. The 22 locations are stratified into three zones: South, Middle, and North (Buntaran et al. 2019). Two of the four new locations are located in the North zone, and two in the South zone. The new locations have no observations of yield for any of the genotypes but do have data on environmental covariates. The layout of the trials at each location was an α-design with two replicates. The available covariates were soil properties, i.e. pH, clay content, and humus content. The covariates are location-specific.

### Statistical models

A two-stage fully-efficient stage-wise approach was used, which forwards the full variance–covariance matrix of adjusted means from stage I (Damesa et al. 2017; Piepho et al. 2012) to the final analysis in stage II of a stage-wise analysis. In stage I, each location was analysed individually using a linear mixed model (LMM). The general equation of an LMM is written as

1 $$ {\mathbf{Y}} = \mathbf{X\beta } + {\mathbf{Zu}} + {\mathbf{e}} $$

where $\mathbf{Y}$ is the vector of yield is the design matrix for fixed-effects of vector ${\varvec{\upbeta}}$, $\mathbf{Z}$ is the incidence matrix for random-effects of vector $\mathbf{u}$, and $\mathbf{e}$ is the vector residuals. The distributions of the random effects and the residuals are $\mathbf{u}\sim N(\mathbf{0},\mathbf{G})$ and $\mathbf{e}\sim N\left(\mathbf{0},\mathbf{R}\right).$ Then, the distribution of the response is $\mathbf{Y}\sim N(\mathbf{X}{\varvec{\upbeta}},\mathbf{V})$, where $\mathbf{V}=\mathbf{Z}\mathbf{G}{\mathbf{Z}}^{\mathbf{^{\prime}}}+\mathbf{R}$.

In our case, in stage I, the fixed effects $\mathbf{X}{\varvec{\upbeta}}$ for the $jm$-th trial are

$$ \mu_{ijm} = \phi_{jm} + \tau_{ijm} $$

where the ${\phi }_{jm}$ is the intercept of the $j$-th location nested in the $m$-th zone, and ${\tau }_{ijm}$ is the effects of $i$-th genotype in the $j$-th location nested in the $m$-th zone, for $i=1, 2,\dots ,I$, $j= 1, 2, \dots , {J}_{m}$, and $m=1, 2,\dots ,M$. The letter $I$ denotes the number of genotypes, $M$ is the number of zones, ${J}_{m}$ is the number of locations within the $m$-th zone, and $J=\sum_{m=1}^{M}{J}_{m}$ is total number of locations. The random effects $\mathbf{Z}\mathbf{u}$ and **e** are

$${r}_{jkm}+{b}_{jklm}+{e}_{ijklm}$$

where ${r}_{jkm}\sim N(0,{\sigma }_{r}^{2})$ is the random effect of the $k$-th replication in the $j$-th location nested in the $m$-th zone, ${b}_{jklm}\sim N(0,{\sigma }_{b}^{2})$ is the random effect of the $s$-th incomplete block of the $k$-th replication in the $j$-th location nested in the $m$-th zone, and ${e}_{ijklm}$ is the residual plot error associated with the observation ${y}_{ijklm}$ in vector $\mathbf{Y}$. In a single-stage analysis, the replication effect is random with necessity because it is nested within location, which is a random factor. Thus, to mimic this approach in the fully efficient two-stage analysis, the replication effect is assigned to be random.

From stage I analysis, we obtained the adjusted means at the $j$-th location in the $m$-th zone, ${\widehat{\mu }}_{ijm}$, which are estimated by best linear unbiased estimation (BLUE), and ${e}_{ijm}$, the error associated with the adjusted means. The adjusted means and the associated error were used for stage II analysis.

In stage II, the analysis was conducted using a model that comprises zone effects since the locations are stratified into three zones. For the stage II model, the vector $\mathbf{Y}$ consists of the means ${\widehat{\mu }}_{ijm}$ from stage I and the error vector $\mathbf{e}$ has sub-vectors ${\mathbf{e}}_{j}$ with elements ${e}_{ijm}$, with $\mathrm{var}\left({\mathbf{e}}_{j}\right)={\mathbf{R}}_{j}$. The variance–covariance matrix ${\mathbf{R}}_{j}$ of adjusted means in the $j$-th location is obtained in stage I by using residual maximum likelihood (REML). The overall variance–covariance matrix of $\mathbf{e}$ is block diagonal, i.e.

$$ {\text{var}} \left( \mathbf{e} \right) = {\mathbf{R}} = \oplus_{j = 1}^{J} {\mathbf{R}}_{j} = \left( {\begin{array}{*{20}c} {{\mathbf{R}}_{1} } & 0 & \cdots & 0 \\ 0 & {{\mathbf{R}}_{2} } & 0 & 0 \\ \vdots & 0 & \ddots & \vdots \\ 0 & 0 & \cdots & {{\mathbf{R}}_{J} } \\ \end{array} } \right) $$

In stage II, seven fixed-genotype-effect (FG) and seven random-genotype-effect (RG) statistical models were fitted. The entries in vectors ${\mathbf{X}}{\mathbf{\beta}}$ and ${\mathbf{Z}}{\mathbf{u}}$ of the FG and RG models are presented in Tables 1, 2, respectively. In stage II, for all 14 models, the location effect nested in the $m$-th zone is random, whereas the effect of the zone, ${\zeta }_{m}$, is fixed. In the RG models, the genotype × zone interaction effect, ${\left(g\zeta \right)}_{im}$, is random because genotype is a random factor. The random genotype main effect and genotype × zone interaction effect allow exploiting information across zones when computing zone-based prediction of genotypes (Buntaran et al. 2019; Kleinknecht et al. 2013; Piepho and Möhring 2005; Piepho et al. 2016).

**Table 1:** The seven fixed-genotype-effect (FG) models

Model	${{\mathbf{X}}\mathbf{\beta} }$	$\mathbf{Zu}$*	Covariance structure
FG1	$\alpha + \zeta_{m} + \tau_{i} + \left( {\tau \zeta } \right)_{im}$	${l}_{jm}+{\left(\tau l\right)}_{ijm}$	${\mathbf{G}}_{l} = \oplus_{m = 1}^{M} {\mathbf{G}}_{{l_{m} }} , {\mathbf{G}}_{{l_{m} }} = \sigma_{{l_{m} }}^{2} {\mathbf{I}}$ ${\varvec{G}}_{\tau l} = \oplus_{m = 1}^{M} {\varvec{G}}_{{(\tau l)_{m} }} ,{\varvec{G}}_{{\left( {\tau l} \right)_{m} }} = \sigma_{{\left( {\tau l} \right)_{m} }}^{2} {\varvec{I}}$
FG2	$\alpha + \zeta_{m} + \tau_{i} + \left( {\tau \zeta } \right)_{im}$	${l}_{jm}+{\left(\tau l\right)}_{ijm}$	${\mathbf{G}}_{l} = \sigma_{l}^{2} {\mathbf{I}}$ ${\mathbf{G}}_{\tau l}={\upsigma }_{\tau l}^{2}\mathbf{I}$
FGC	$\alpha +\beta {x}_{jm}+{\zeta }_{m} + \tau_i + \left( {\tau \zeta } \right)_{im} + {\lambda }_{m}{x}_{jm}$	${l}_{jm}+{\left(\tau l\right)}_{ijm}$	${\mathbf{G}}_{l} = \oplus_{m = 1}^{M} {\mathbf{G}}_{{l_{m} }} , {\mathbf{G}}_{{l_{m} }} = \sigma_{{l_{m} }}^{2} {\mathbf{I}}$ ${\mathbf{G}}_{\tau l} = \oplus_{m = 1}^{M} {\mathbf{G}}_{{(\tau l)_{m} }} ,{\mathbf{G}}_{{\left( {\tau l} \right)_{m} }} = \sigma_{{\left( {\tau l} \right)_{m} }}^{2} {\mathbf{I}}$
FGCQ	$\alpha +\beta {x}_{jm}+\gamma {x}_{jm}^{2}+{\zeta }_{m}+ \tau_i + (\tau\zeta)_{im} + {\lambda }_{m}{x}_{jm}$	${l}_{jm}+{\left(\tau l\right)}_{ijm}$	${\mathbf{G}}_{l} = \oplus_{m = 1}^{M} {\mathbf{G}}_{{l_{m} }} , {\mathbf{G}}_{{l_{m} }} = \sigma_{{l_{m} }}^{2} {\mathbf{I}}$ ${\mathbf{G}}_{\tau l} = \oplus_{m = 1}^{M} {\mathbf{G}}_{{(\tau l)_{m} }} ,{\mathbf{G}}_{{\left( {\tau l} \right)_{m} }} = \sigma_{{\left( {\tau l} \right)_{m} }}^{2} {\mathbf{I}}$
FGI1	$$\begin{gathered} \alpha + \beta_{1} x_{jm} + \gamma x_{jm}^{2} + \hfill \\ \zeta_{m} + \lambda_{m} x_{jm} + \hfill \\ \left( {\tau_{i} + \beta_{2i} x_{jm} + \beta_{3i} x_{jm}^{2} } \right) + \hfill \\ \left( {\left( {\tau \zeta } \right)_{im} + \beta_{{4_{im} }} x_{jm} + \beta_{{5_{im} }} x_{jm}^{2} } \right) \hfill \\ \end{gathered}$$	${l}_{jm}+{\left(\tau l\right)}_{ijm}$	${\mathbf{G}}_{l} = \oplus_{m = 1}^{M} {\mathbf{G}}_{{l_{m} }} , {\mathbf{G}}_{{l_{m} }} = \sigma_{{l_{m} }}^{2} {\mathbf{I}}$ ${\mathbf{G}}_{\tau l} = \oplus_{m = 1}^{M} {\mathbf{G}}_{{(\tau l)_{m} }} ,{\mathbf{G}}_{{\left( {\tau l} \right)_{m} }} = \sigma_{{\left( {\tau l} \right)_{m} }}^{2} {\mathbf{I}}$
FGI2	$$\begin{gathered} \alpha + \beta x_{jm} + \gamma x_{jm}^{2} + \zeta_{m} + \tau_{i} + \lambda_{m} x_{jm} \hfill \\ + \left( {\left( {\tau \zeta } \right)_{im} + \beta_{{4_{im} }} x_{jm} + \beta_{{5_{im} }} x_{jm}^{2} } \right) \hfill \\ \end{gathered}$$	$${l}_{jm}+{\left(\tau l\right)}_{ijm}$$	$${\mathbf{G}}_{l} = \oplus_{m = 1}^{M} {\mathbf{G}}_{{l_{m} }} , {\mathbf{G}}_{{l_{m} }} = \sigma_{{l_{m} }}^{2} {\mathbf{I}}$$$${\mathbf{G}}_{\tau l} = \oplus_{m = 1}^{M} {\mathbf{G}}_{{(\tau l)_{m} }} ,{\mathbf{G}}_{{\left( {\tau l} \right)_{m} }} = \sigma_{{\left( {\tau l} \right)_{m} }}^{2} {\mathbf{I}}$$
FGI3	$$\begin{gathered} \alpha + \beta x_{jm} + \hfill \\ \gamma x_{jm}^{2} + \zeta_{m} + \left( {\tau \zeta } \right)_{im} + \lambda_{m} x_{jm} \hfill \\ + \left( {\tau_{i} + \beta_{2i} x_{jm} + \beta_{3i} x_{jm}^{2} } \right) \hfill \\ \end{gathered}$$	${l}_{jm}+{\left(\tau l\right)}_{ijm}$	$$\begin{gathered} {\mathbf{G}}_{l} = \oplus_{(m = 1)}^{M} \mathbf{G}_{{(l_{m} )}} ,\mathbf{G}_{{(l_{m} )}} = \sigma_{{(l_{m} )}}^{2} \text {I} \hfill \\ \end{gathered}$$ $$\begin{gathered} {\mathbf{G}}_{\tau l} \hfill = \oplus_{m = 1}^{M} {\mathbf{G}}_{{(\tau l)_{m} }} ,{\mathbf{G}}_{{\left( {\tau l} \right)_{m} }} = \sigma_{{\left( {\tau l} \right)_{m} }}^{2} {\mathbf{I}} \hfill \\ \end{gathered}$$

*The index *i* refers to the *i*-th genotype, *j* refers to the *j*-th location, and *m* refers to the *m*-th zone

**Table 2:** The seven random-genotype effect (RG) models

Model	${{\mathbf{X}}{\mathbf\beta} }$	$\mathbf{Zu}$*	Covariance structure
RG1	$\alpha +{\zeta }_{m}$	${l}_{jm}+ {g}_{i}+{\left(g\zeta \right)}_{im}+{\left(gl\right)}_{ijm}$	${\mathbf{G}}_{l} = \oplus_{m = 1}^{M} {\mathbf{G}}_{{l_{m} }} , {\mathbf{G}}_{{l_{m} }} = \sigma_{{l_{m} }}^{2} {\mathbf{I}}$ ${\mathbf{G}}_{g}={\upsigma }_{g}^{2}\mathbf{I}$ ${\mathbf{G}}_{g\zeta }={\upsigma }_{g\zeta }^{2}\mathbf{I}$ ${\mathbf{G}}_{gl} = \oplus_{m = 1}^{M} {\mathbf{G}}_{{(gl)_{m} }} ,{\mathbf{G}}_{{\left( {gl} \right)_{m} }} = \sigma_{{\left( {gl} \right)_{m} }}^{2} {\mathbf{I}}$
RG2	$\alpha +{\zeta }_{m}$	${l}_{jm}+ {g}_{i}+{\left(g\zeta \right)}_{im}+{\left(gl\right)}_{ijm}$	${\mathbf{G}}_{l}={\upsigma }_{l}^{2}\mathbf{I}$ ${\mathbf{G}}_{g}={\upsigma }_{g}^{2}\mathbf{I}$ ${\mathbf{G}}_{g\zeta }={\upsigma }_{g\zeta }^{2}\mathbf{I}$ ${\mathbf{G}}_{gl}={\upsigma }_{gl}^{2}\mathbf{I}$
RGC	$\alpha +\beta {x}_{jm}+{\zeta }_{m}+{\lambda }_{m}{x}_{jm}$	${l}_{jm}+ {g}_{i}+{\left(g\zeta \right)}_{im}+{\left(gl\right)}_{ijm}$	${\mathbf{G}}_{{l_{jm} }} = \oplus_{m = 1}^{M} {\mathbf{G}}_{{l_{m} }} , {\mathbf{G}}_{{l_{m} }} = \sigma_{{l_{m} }}^{2} {\mathbf{I}}$ ${\mathbf{G}}_{{g}_{i}}={\upsigma }_{g}^{2}\mathbf{I}$ ${\mathbf{G}}_{g\zeta }={\upsigma }_{g\zeta }^{2}\mathbf{I}$ ${\mathbf{G}}_{gl} = \oplus_{m = 1}^{M} {\mathbf{G}}_{{(gl)_{m} }} ,{\mathbf{G}}_{{\left( {gl} \right)_{m} }} = \sigma_{{\left( {gl} \right)_{m} }}^{2} {\mathbf{I}}$
RGCQ	$\alpha +\beta {x}_{jm}+\gamma {x}_{jm}^{2}+{\zeta }_{m}+{\lambda }_{m}{x}_{jm}$	${l}_{jm}+ {g}_{i}+{\left(g\zeta \right)}_{im}+{\left(gl\right)}_{ijm}$	$\mathbf{G}_{l} = \oplus_{m = 1}^{M} \mathbf{G}_{{l_{m} }} , \mathbf{G}_{{l_{m} }} = \sigma_{{l_{m} }}^{2} \mathbf{I}$ ${\mathbf{G}}_{g}={\upsigma }_{g}^{2}\mathbf{I}$ ${\mathbf{G}}_{g\zeta }={\upsigma }_{g\zeta }^{2}\mathbf{I}$ $\mathbf{G}_{gl} = \oplus_{m = 1}^{M} \mathbf{G}_{{(gl)_{m} }} ,\mathbf{G}_{{\left( {gl} \right)_{m} }} = \sigma_{{\left( {gl} \right)_{m} }}^{2} \mathbf{I}$
RC1	$\alpha +\beta {x}_{jm}+\gamma {x}_{jm}^{2}+{\zeta }_{m}+{\lambda }_{m}{x}_{jm}$	${l}_{jm}+\left({a}_{i}+{b}_{i}{x}_{jm}+{c}_{i}{x}_{jm}^{2}\right)$ $+\left({d}_{im}+{h}_{im}{x}_{jm}+{p}_{im}{x}_{jm}^{2}\right)$ $+{\left(gl\right)}_{ijm}$	$\mathbf{G}_{l} = \oplus_{m = 1}^{M} \mathbf{G}_{{l_{m} }} , \mathbf{G}_{{l_{m} }} = \sigma_{{l_{m} }}^{2} \mathbf{I}$ $$\left[\begin{array}{c}{a}_{i}\\ {b}_{i}\\ {c}_{i}\end{array}\right]\sim iid\, N\left(\mathbf{0},{\mathbf{G}}_{{g}_{i}}\right)$$ $${\mathbf{G}}_{g}=\left[\begin{array}{ccc}{\sigma }_{a}^{2}& {\sigma }_{ab}& {\sigma }_{ac}\\ {\sigma }_{ab}& {\sigma }_{b}^{2}& {\sigma }_{bc}\\ {\sigma }_{ac}& {\sigma }_{bc}& {\sigma }_{c}^{2}\end{array}\right]$$
			$$\left[\begin{array}{c}{d}_{im}\\ {h}_{im}\\ {p}_{im}\end{array}\right]\sim iid\, N\left(\mathbf{0},{\mathbf{G}}_{{\left(g\zeta \right)}_{im}}\right)$$ $${\mathbf{G}}_{g\zeta }=\left[\begin{array}{ccc}{\sigma }_{d}^{2}& {\sigma }_{dh}& {\sigma }_{dp}\\ {\sigma }_{dh}& {\sigma }_{h}^{2}& {\sigma }_{hp}\\ {\sigma }_{dp}& {\sigma }_{hp}& {\sigma }_{p}^{2}\end{array}\right]$$ $\mathbf{G}_{gl} = \oplus_{m = 1}^{M} \mathbf{G}_{{(gl)_{m} }} ,\mathbf{G}_{{\left( {gl} \right)_{m} }} = \sigma_{{\left( {gl} \right)_{m} }}^{2} \mathbf{I}$
RC2	$\alpha +\beta {x}_{jm}+\gamma {x}_{jm}^{2}+{\zeta }_{m}+{\lambda }_{m}{x}_{jm}$	${l}_{jm}+{g}_{i}$ $+\left({d}_{im}+{h}_{im}{x}_{jm}+{p}_{im}{x}_{jm}^{2}\right)$ $+{\left(gl\right)}_{ijm}$	$\mathbf{G}_{l} = \oplus_{m = 1}^{M} \mathbf{G}_{{l_{m} }} , \mathbf{G}_{{l_{m} }} = \sigma_{{l_{m} }}^{2} \mathbf{I}$ ${\mathbf{G}}_{g}={\upsigma}_{{g}}^{2}\mathbf{I}$ $$\left[\begin{array}{c}{d}_{im}\\ {h}_{im}\\ {p}_{im}\end{array}\right]\sim iid\, N\left(\mathbf{0},{\mathbf{G}}_{{\left(g\zeta \right)}_{im}}\right)$$ $${\mathbf{G}}_{{\left(g\zeta \right)}_{im}}=\left[\begin{array}{ccc}{\sigma }_{d}^{2}& {\sigma }_{dh}& {\sigma }_{dp}\\ {\sigma }_{dh}& {\sigma }_{h}^{2}& {\sigma }_{hp}\\ {\sigma }_{dp}& {\sigma }_{hp}& {\sigma }_{p}^{2}\end{array}\right]$$ $\mathbf{G}_{gl} = \oplus_{m = 1}^{M} \mathbf{G}_{{(gl)_{m} }} ,\mathbf{G}_{{\left( {gl} \right)_{m} }} = \sigma_{{\left( {gl} \right)_{m} }}^{2} \mathbf{I}$
RC3	$\alpha +\beta {x}_{jm}+\gamma {x}_{jm}^{2}+{\zeta }_{m}+{\lambda }_{m}{x}_{jm}$	${l}_{jm}+\left({a}_{i}+{b}_{i}{x}_{jm}+{c}_{i}{x}_{jm}^{2}\right)$ $+{\left(g\zeta \right)}_{im}+{\left(gl\right)}_{ijm}$	$\mathbf{G}_{l} = \oplus_{m = 1}^{M} \mathbf{G}_{{l_{m} }} , \mathbf{G}_{{l_{m} }} = \sigma_{{l_{m} }}^{2} \mathbf{I}$ ${\mathbf{G}}_{g\zeta}={\upsigma }_{g\zeta}^{2}\mathbf{I}$ $$\left[\begin{array}{c}{a}_{i}\\ {b}_{i}\\ {c}_{i}\end{array}\right]\sim iid\, N\left(\mathbf{0},{\mathbf{G}}_{{g}_{i}}\right)$$ $${\mathbf{G}}_{g}=\left[\begin{array}{ccc}{\sigma }_{a}^{2}{\sigma }_{ab} {\sigma }_{ac}\\ {\sigma }_{ab} {\sigma }_{b}^{2} {\sigma }_{bc}\\ {\sigma }_{ac} {\sigma }_{bc} {\sigma }_{c}^{2}\end{array}\right]$$

*The index *i* refers to the *i*-th genotype, *j* refers to the *j*-th location, and *m* refers to the *m*-th zone

In the seven FG models, two models are LMM without covariates but with different variance–covariance structure for location and genotype × location effects, two models are LMM with covariate modelled by fixed effects, and three models are LMM with covariate interactions terms. Below, we describe each model in Table 1:

1. FG1 is the baseline LMM without covariate. The random-effect terms in this model are the effect of location nested in the $m$-th zone, ${l}_{jm}\sim N(0,{\sigma }_{{l}_{m}}^{2})$, and the interaction effect of genotype and location nested in the $m$-th zone. ${\left(\tau l\right)}_{ijm}\sim N(0,{\sigma }_{(\tau{l)}_{m}}^{2})$. The variance–covariance structure for the location effect, ${l}_{jm}$, is a heterogeneous zone-specific covariance structure, ${\mathbf{G}}_{l} = \oplus_{m = 1}^{M} {\mathbf{G}}_{{l_{m} }}$, where ${\mathbf{G}}_{{l}_{m}}={\sigma }_{{l}_{m}}^{2}\mathbf{I}$ with **I** a ${J}_{m}$-dimensional identity matrix. The structure for the genotype × location effect, ${\left(g l\right)}_{ijm}$, is also a heterogeneous zone-specific covariance structure is ${\mathbf{G}}_{gl} = \oplus_{m = 1}^{M} {\mathbf{G}}_{{(gl)_{m} }}$, where ${\mathbf{G}}_{(g {l)}_{m}}$ is the ${J}_{m}I$-dimensional diagonal matrix ${\sigma }_{(g {l)}_{m}}^{2}\mathbf{I}$, assuming that all genotypes were tested in all locations.
2. FG2 is the FG1 model but with homogeneous variance–covariance structure for the location and genotype × location effects. Thus, the location effect, ${l}_{jm}$ has the *J*-dimensional structure ${\mathbf{G}}_{l}={\upsigma }_{l}^{2}\mathbf{I}$, and for the genotype × location effect ${\left(g l\right)}_{ijm}$, the $JI$-dimensional structure is ${\upsigma }_{g l}^{2}\mathbf{I}$.
3. FGC is a model with a covariate. The covariate values, ${x}_{jm}$, are location-specific. In this model, the covariate is modelled by a linear trend. The notation for fixed regression terms involving the covariate is $\beta {x}_{jm}+{\lambda }_{m}{x}_{jm}$, where $\beta$ is the fixed effect for the slope of the covariate and ${\lambda }_{m}$ is the fixed effect for the zone-specific slope of the covariate for the $m$-th zone. The variance–covariance structures for genotype, location, genotype × zone, and genotype × location effects are the same as in the FG1 model.
4. FGCQ is the FGC model with an additional quadratic term for the covariate, ${x}_{jm}^{2}$. The slope for this quadratic term is denoted as $\gamma$. Note that in this model, the zone-specific interaction term of the quadratic covariate was not included because it was not significant. Thus, we decided to settle for the FGCQ model without the zone-specific quadratic term.
5. FGI1 is a model with covariate interactions terms for genotype, ${\tau}_{i}$, and genotype × zone, ${\left(\tau\zeta \right)}_{im}$. Hence, this model has genotype and genotype × zone specific coefficients for the intercepts and slopes. FGI1 is the most complex model because it has linear and quadratic terms for the covariate interacting with genotype and genotype × zone. The model for covariate interaction in the genotype term is $\left({\tau }_{i}+{{\beta }_{2}}_{i}{x}_{jm}+{\beta }_{{3}_{i}}{x}_{jm}^{2}\right)$ where ${\tau }_{i}$ is the genotype-specific intercept for the $i$-th genotype, ${{\beta }_{2}}_{i}$ is the linear genotype-specific slope for the $i$-th genotype, and ${{\beta }_{3}}_{i}$ is the quadratic genotype-specific slope for the $i$-th genotype. The model for covariate interaction in the genotype × zone term is $\left({(\tau \zeta )}_{im}+{\beta }_{{4}_{im}}{x}_{jm}+{\beta }_{{5}_{im}}{x}_{jm}^{2}\right)$, where ${(\tau \zeta )}_{im}$ is the genotype × zone-specific intercept for the $i$-th genotype in the $m$-th zone, ${{\beta }_{4}}_{im}$ is the linear genotype × zone-specific slope for the $i$-th genotype in the $m$-th zone, and ${{\beta }_{5}}_{im}$ is the quadratic genotype × zone-specific slope for the $i$-th genotype in the $m$-th zone.
6. FGI2 is a reduced version of the FGI1 model. The regression coefficients for the linear and quadratic term in the genotype main effect are removed.
7. FGI3 is a reduced version of the FGI1 model. The regression coefficients for the linear and quadratic term in the genotype × zone term are removed.

For the seven RG models, the differences compared to the seven FG models is that the genotype effect was random and there were three RC models due to the interactions between the covariate and genotype and genotype × zone effects. In the seven RG models, two models are LMM without covariates but with different variance–covariance structures for location and genotype × location effects, two models are LMM with covariate modelled by fixed effects, and three models are LMM with random coefficients for the regression on covariates. Below, we describe each model in Table 2:

1. RG1 is the baseline LMM without covariate and random coefficients. The random-effect terms in this model are the effect of genotype, ${g}_{i}\sim N(0,{\sigma }_{g}^{2})$, the effect of location nested in the $m$-th zone, ${l}_{jm}\sim N(0,{\sigma }_{{l}_{m}}^{2})$, the interaction effect of genotype and zone, ${\left(g\zeta \right)}_{im}\sim N(0,{\sigma }_{g\zeta }^{2})$, and the interaction effect of genotype and location nested in the $m$-th zone ${\left(gl\right)}_{ijm}\sim N(0,{\sigma }_{{\left(gl\right)}_{m}}^{2})$. The variance–covariance structure for the genotype effect, ${g}_{i}$, is ${\mathbf{G}}_{g}={\upsigma }_{g}^{2}\mathbf{I}$. The structure for the genotype × zone effect, ${\left(g\zeta \right)}_{im}$, is ${\mathbf{G}}_{g\zeta }={\upsigma }_{g\zeta }^{2}\mathbf{I}$. For the location effect, ${l}_{jm}$, the variance–covariance structure is a heterogeneous zone-specific covariance structure, heterogeneous zone-specific covariance structure, ${\mathbf{G}}_{l} = \oplus_{m = 1}^{M} {\mathbf{G}}_{{l_{m} }}$, where ${\mathbf{G}}_{{l}_{m}}={\sigma }_{{l}_{m}}^{2}\mathbf{I}$ with **I** a ${J}_{m}$-dimensional identity matrix. The structure for the genotype × location effect, ${\left(\tau l\right)}_{ijm}$, is also a heterogeneous zone-specific covariance structure is ${\mathbf{G}}_{\tau 1} = \otimes_{m = 1}^{M} {\mathbf{G}}_{{(\tau l)_{m} }}$, where ${\mathbf{G}}_{(\tau {l)}_{m}}$ is the ${J}_{m}I$-dimensional diagonal matrix ${\sigma }_{(\tau {l)}_{m}}^{2}\mathbf{I}$, assuming that all genotypes were tested in all locations.
2. RG2 is the RG1 model but with homogeneous variance–covariance structure for the location and genotype × location effects. Thus, the location effect, ${l}_{jm}$ has the *J*-dimensional structure ${\mathbf{G}}_{l}={\upsigma }_{l}^{2}\mathbf{I}$, and for the genotype × location effect ${\left(\tau l\right)}_{ijm}$, the $JI$-dimensional structure is ${\upsigma }_{g l}^{2}\mathbf{I}$.
3. RGC is a model with a covariate. The covariate values, ${x}_{jm}$, are location-specific. In this model, the covariate is modelled by a linear trend. The notation for the fixed regression terms involving the covariate is $\beta {x}_{jm}+{\lambda }_{m}{x}_{jm}$, where $\beta$ is the fixed effect for the slope of the covariate and ${\lambda }_{m}$ is the fixed effect for the zone-specific slope of the covariate for the $m$-th zone. The variance–covariance structures for the genotype, location, genotype × zone, and genotype × location effects are the same as in the RG1 model. Thus, the random effects comprise no regression terms.
4. RGCQ is the RGC model with an additional quadratic term for the covariate, ${x}_{jm}^{2}$. The slope for this quadratic term is denoted as $\gamma$. As in the FGCQ model, the zone-specific interaction term of the quadratic covariate was not included because it was not significant. Thus, we decided to settle for the RGCQ model without the zone-specific quadratic term.
5. RC1 is a model with random coefficients. Since the covariate is location-specific, it was not possible to fit random coefficients for location. Thus, a RC model was fitted for the genotype term, ${g}_{i}$, and the genotype × zone term, ${\left(g\zeta \right)}_{im}$. Hence, this model has geno type- and genotype × zone-specific coefficients for the intercepts and slopes. RC1 is the most complex model because it has linear and quadratic terms for the covariate, and allows a covariance between intercept and slope effects, meaning that ${\mathbf{G}}_{g}$ and ${\mathbf{G}}_{g\zeta }$ are unstructured variance–covariance matrices. It is essential to allow the covariance between intercept and slope to be a free parameter in order to ensure invariance with respect to translation and scale transformation of the covariates (Longford 1993; Piepho and Ogutu 2002). Note that when the covariance structure is diagonal, then the structure is only invariant to scale transformations but not to translations (Wolfinger 1996). The model for random coefficients in the genotype term was ${g}_{i}={a}_{i}+{b}_{i}{x}_{jm}+{c}_{i}{x}_{jm}^{2}$, where ${a}_{i}$ is the random genotype-specific intercept for the $i$-th genotype, ${b}_{i}$ is the random linear genotype-specific slope for the $i$-th genotype, and ${c}_{i}$ is the random quadratic genotype-specific slope for the $i$-th genotype. The model for random coefficients in the genotype × zone term was ${\left(g\zeta \right)}_{im}={d}_{im}+{h}_{im}{x}_{jm}+{p}_{im}{x}_{jm}^{2}$, where ${d}_{im}$ is the random genotype × zone specific intercept for the $i$-th genotype in the $m$-th zone, ${h}_{im}$ is the random linear genotype × zone-specific slope for the $i$-th genotype in the $m$-th zone, and ${c}_{i}$ is the random quadratic genotype × zone-specific slope for the $i$-th genotype in the $m$-th zone.
6. RC2 is a reduced version of model of RC1. The random regression coefficients for the linear and quadratic terms in the genotype main effect are dropped completely, so its covariance structure is ${\mathbf{G}}_{g}={\upsigma }_{g}^{2}\mathbf{I}$.
7. In the RC3 model, only the random coefficients for the genotype × zone term is removed completely, so its covariance structure is ${\mathbf{G}}_{g\zeta }={\upsigma }_{g\zeta }^{2}\mathbf{I}$.

### Covariate selection and scaling

The covariate selection was done by extending the fixed-effects part of the FG1 model with the covariates as follows:

$$\alpha +{\zeta }_{m}+{\tau }_{i}+{\left(\tau \zeta \right)}_{im}+{\beta }_{1}{{x}_{1}}_{jm}+{\beta }_{2}{{x}_{2}}_{jm}+{\beta }_{3}{{x}_{3}}_{jm}+{\beta }_{4}{{x}_{4}}_{jm}+{\beta }_{5}{{x}_{5}}_{jm}+{\beta }_{6}{{x}_{6}}_{jm}$$

Parameters ${\beta }_{1}$ and ${\beta }_{2}$ are the coefficients of linear and quadratic terms for the scaled clay covariate, respectively, ${\beta }_{3}$ and ${\beta }_{4}$ are the coefficients of linear and quadratic terms for the scaled pH covariate, respectively, and ${\beta }_{5}$ and ${\beta }_{6}$ are the coefficients of linear and quadratic terms for the scaled humus covariate, respectively. The pH and humus covariates were standardised to mean 0 and standard deviation 1. The clay covariate was scaled as $(\mathrm{clay}-40)/10$ since this scaling resulted in non-negative variance estimates for the RC1 model. The fixed effect tests were adjusted with Kenward-Roger denominator degrees of freedom (Kenward and Roger 1997). If the F-test of a covariate effect was not significant at α = 5%, the covariate was dropped. The quadratic terms were tested first. The linear terms were tested linear only if the quadratic had to be dropped.

### Predictions of genotypes in new locations

All models in Tables 1 and 2, which are special cases of Eq. 1, were fitted using the mixed model equations (MME) in Eq. 2 (Henderson 1950). The information used to estimate the **G** and **R** matrices in this MME are the records from the tested locations. Since the **G** and **R** matrices are unknown, they are estimated from the data via REML, producing estimates $\widehat{\mathbf{G}}$ and $\widehat{\mathbf{R}}$:

2 $$ \left[ {\begin{array}{*{20}c} {\mathbf{{X^{\prime}\hat{R}}}^{ - 1} {\mathbf{X}}} & {\mathbf{{X^{\prime}\hat{R}}}^{ - 1} {\mathbf{Z}}} \\ {\mathbf{{Z^{\prime}\hat{R}}}^{ - 1} {\mathbf{X}}} & {\mathbf{{Z^{\prime}\hat{R}}}^{ - 1} {\mathbf{Z}} + \hat{\mathbf{{G}}}^{ - 1} } \\ \end{array} } \right]\left[ {\begin{array}{*{20}c} {\hat{\mathbf{\beta }}} \\ {\hat{\mathbf{u}}} \\ \end{array} } \right] = \left[ {\begin{array}{*{20}c} {\mathbf{{X^{\prime}\hat{R}}}^{ - 1} {\mathbf{y}}} \\ {\mathbf{{Z^{\prime}\hat{R}}}^{ - 1} {\mathbf{y}}} \\ \end{array} } \right] $$

The solution for $\widehat{{\mathbf{\beta}}}$, which is the empirical BLUE (EBLUE), is obtained via generalised least-squares

3 $${\left({{\mathbf{X}}}^{{\prime}}{\widehat{\mathbf{V}}}^{-1}{\mathbf{X}}\right)}^{-1}{{\mathbf{X}}}^{{{\prime}}}{\widehat{\mathbf{V}}}^{-1}{\mathbf{y}}$$

and $\widehat{\mathbf{u}}$, the empirical best linear unbiased predictor (EBLUP) of $\mathbf{u}$, is

4 $$\widehat{{\mathbf{G}}}{{\mathbf{Z}}}^{\boldsymbol{^{\prime}}}{\widehat{\mathbf{V}}}^{-1}({\mathbf{y}}-{\mathbf{X}}\widehat{{\mathbf{\beta}}})$$

McLean et al. (1991) discussed three types of inference space, i.e. the broad, narrow, and intermediate spaces. Estimates for these inference spaces are computed based on estimable functions ${\mathbf{K}}^{\mathbf{^{\prime}}}{\mathbf{\beta}}$, which are linear combinations of fixed effects only, and predictable functions ${\mathbf{K}}^{\mathbf{^{\prime}}}{\mathbf{\beta}}+\mathbf{M}\mathbf{^{\prime}}\mathbf{u}$, which consist of linear combinations of fixed effects and random effects $\mathbf{M}\mathbf{^{\prime}}\mathbf{u}$. The matrix $\mathbf{K}\mathbf{^{\prime}}$ consists of coefficients 1 and 0, where 1 indicates fixed effects needed in the estimable function, and covariate values for models that include covariates. The matrix $\mathbf{M}\mathbf{^{\prime}}$ contains the coefficients 1 and 0 according to the relevant random effects in the predictable function, and covariates for the RC models. The estimable function results in the BLUE, while the predictable function results in the BLUP. The differences between three types of inference space are explained in the Appendix.

In this study, the intermediate inference space (McLean et al 1991) is useful because it determines which genotype is the best in the particular environment. Hence, we focus on this inference space. The predictable functions for the EBLUPs in the new location can be expressed as

5 $$w=\mathbf{K}\mathbf{^{\prime}}{\mathbf{\beta}}+\mathbf{M}\mathbf{^{\prime}}\mathbf{u}+{\mathbf{M}}_{0}^{\mathbf{^{\prime}}}{\mathbf{u}}_{0}$$

where $\mathbf{K}\mathbf{^{\prime}}{\mathbf{\beta}}$ is the estimable function of fixed effects for zone. This term also includes fixed covariate terms for the models that use covariates. The matrix $\mathbf{K}\mathbf{^{\prime}}$ selects the fixed effects for the targeted zone where the new location is located, and the covariates for models with covariate. The term $\mathbf{M}\mathbf{^{\prime}}\mathbf{u}$ is the predictable function involving the genotype main effect and the genotype × zone interaction effects of the zone where the new location located, as well as any random covariate terms. The matrix $\mathbf{M}\mathbf{^{\prime}}$ selects the relevant random effects that can be estimated from the tested location; thus, it comprises the main effects of genotype and the genotype × zone interactions. For RC models, the matrix ${\mathbf{M}}^{\mathbf{^{\prime}}}$ includes intercept and slope for the random-effects terms that have interactions with covariates. The last term, ${\mathbf{M}}_{0}^{\mathbf{^{\prime}}}{\mathbf{u}}_{0}$, is the predictable function of the location main effect and genotype × location interaction for the new location, with $\mathrm{var}({\mathbf{u}}_{0})={\mathbf{G}}_{0}$. The matrix $\mathbf{M}\mathbf{^{\prime}}$ includes any covariate terms for the RC models. In the FG models, there is no $\mathbf{M}\mathbf{^{\prime}}{\mathbf{u}}$ term because the genotype and zone effects are fixed, and their interactions effects are also fixed.

The prediction of ${\mathbf{M}}_{0}^{\mathbf{^{\prime}}}{\mathbf{u}}_{0}$ is always zero because there is no information on these effects for the new location. Thus, the random effect ${\mathbf{u}}_{0}$ is not estimated as such, but its distribution is needed in order to obtain the standard errors of predictions of genotypic values (SEPV) and standard errors of the predictions of pairwise differences of genotypic values (SEPD) for the genotypes in the new location. An important point to be observed here is that the random effects ${\mathbf{u}}_{0}$ for a new location are stochastically independent of the BLUP of $\mathbf{K}\mathbf{^{\prime}}{\mathbf{\beta}}+\mathbf{M}\mathbf{^{\prime}}\mathbf{u}$, which can be regarded as the conditional expectation of $w$, given fixed and random effects of the model for the observed data. Thus, the estimable effects can be expressed as a conditional mean as follows:

6 $$\eta =E\left(w|{\mathbf{\beta}},\mathbf{u}\right)={\mathbf{K}}^{\mathbf{^{\prime}}}{\mathbf{\beta}}+{\mathbf{M}}^{\mathbf{^{\prime}}}\mathbf{u}$$

Furthermore, on account of the independence assumption, $\mathrm{var}\left(\mathbf{u}\right)=\mathbf{G}$, $\mathrm{var}\left({\mathbf{u}}_{0}\right)={\mathbf{G}}_{0}$, and $\mathrm{var}\left(\left[\mathbf{u},{\mathbf{u}}_{0}\right]\right)=\mathrm{diag}(\mathbf{G}, {\mathbf{G}}_{0})$, the conditional variance of $w$ is:

7 $$\mathrm{var}\left(w|{\mathbf{\beta}},\mathbf{u}\right)=\mathrm{var}({\mathbf{M}}_{0}^{\mathbf{^{\prime}}}{\mathbf{u}}_{0})={{\mathbf{M}}_{0}^{\mathbf{^{\prime}}}{\mathbf{G}}_{0}\mathbf{M}}_{0}$$

## Precision measures: SEPV, SEPD, and prediction intervals

### SEPV and SEPD in new locations

In the further derivation, it is key to observe that the estimate of $\eta$ in (6) and the random effects ${u}_{0}$ for the new locations are stochastically independent. Thus, the total variance of the prediction of $w$ in (5) is simply the sum of the variance of the estimate of $\eta$ in (6) and the variance in (7). Hence, the SEPV in the new locations are computed as follows:

8 $$SEPV=\sqrt{\mathrm{var}\left(\widehat{\eta }\right)+\mathrm{var}\left(w|{\mathbf{\beta}},\mathbf{u}\right)}$$

where $\mathrm{var}\left(\widehat{\eta }\right)$ is the square of standard errors of EBLUPs for zone-based genotype average, and for our models $\mathrm{var}\left(w|{\mathbf{\beta}},\mathbf{u}\right)$ is the sum of the variance components of effects for location and genotype × location. For the models with covariates, $\mathrm{var}\left(w|{\mathbf{\beta}},\mathbf{u}\right)$ includes the variances and covariances for intercepts and slopes of random coefficients. In the FG models, $\mathrm{var}\left(\widehat{\eta }\right)$ is the square of the standard errors of the EBLUE of $\eta$ for the zone-based genotype average.

For plant breeders and growers, the differences between genotypes are more informative than point estimates for individual genotypes. Thus, the pairwise prediction of differences and the SEPD needs to be computed. The predictions of pairwise differences can be computed using coefficients 1 and -1 in the $\mathbf{K}$ and $\mathbf{M}$ matrices according to the difference of interest genotypes.

The SEPD for the new locations are computed as:

9 $$SEPD=\sqrt{VDIFF\left(\widehat{\eta }\right)+2\times {\sigma }_{gl}^{2}}$$

where $VDIFF\left(\widehat{\eta }\right)$ is the square of the standard error of a difference of EBLUPs or EBLUEs for zone-based genotype averages, and ${\sigma }_{gl}^{2}$ is the variance component of genotype × location effects. In the FG models the ${\sigma }_{gl}^{2}$ refers to ${\sigma }_{\tau l}^{2}$. In (9), the latter is assumed homogeneous between zones, as in models FG2 and RG2. Alternatively, the variance may be assumed zone-specific, as in the other models listed in Tables 1 and 2.

### Prediction intervals for the genotypes in the new locations

Meeker et al. (2017) define a prediction interval for a single future observation is an interval that will, with a specified degree of confidence, cover a future randomly selected observation from a distribution with pre-specified coverage probability $(1-\alpha )$. Following this definition, the prediction intervals for genotype performances are regarded as a prediction interval to contain a single future observation. The important assumption of this interval is that the previously sampled locations and the future one can be regarded as random samples from the same distribution. The genotype × location effects for the new location can be regarded as a random sample from the same distribution as that of the genotype × location effects for locations in the dataset. The prediction interval for *w* is centred at $\eta$ and the approximate $(1-\alpha )\times 100\mathrm{\%}$ prediction interval is given by:

10 $$\widehat{\eta }\pm {z}_{1-\alpha /2 }\mathrm{SEPV}$$

where ${z}_{1-\alpha /2}$ is the $(1-\alpha /2 )\times 100\mathrm{\%}$ quantile of the standard normal distribution. The prediction interval for pairwise differences between the $i$-th and $i^{\prime}$-th genotype is given by:

11 $$ DIFF(\hat{\eta }) \pm z_{1 - \alpha /2} SEPD $$

where $\mathrm{DIFF}(\widehat{\eta })$ is the difference in predictions between two genotypes.

## Accuracy measure

### Cross-validation for model selection

A CV study can measure the prediction errors of the model using the mean squared error of prediction (MSEP) of difference. We conducted a leave-one-out CV for model comparison and selection. To mimic the prediction for new locations, we left one location out at a time and assigned its data as the validation set, and the data from the remaining locations as the training set. For the models with covariate, the covariate in the validation set was used for predictions. The MSEP was computed similarly to the MSEP proposed by Piepho (1998) for measuring the prediction accuracy of the models. The MSEP is a standard statistic for evaluating predictive accuracy. Let $y$ and $z$ denote the observed and predicted values, respectively, and let $I$ denote the total number of genotypes, and $J$ the total number of locations. The assessment was measured based on the discrepancies between observed (${y}_{ij}-{y}_{{i}^{^{\prime}}j}$) and predicted (${z}_{ij}-{z}_{{i}^{^{\prime}}j})$ pairwise differences, since the main interest in cultivar trials is in prediction of differences between genotypes rather than performance of individual genotype (Piepho, 1998). The discrepancies between the observed and predicted pairwise differences from the 18 folds were accumulated and the MSEP was computed from this accumulation. The MSEP is computed as follows:

$$MSEP=\frac{{\sum }_{j=1}^{J}\sum_{i=1}^{I}\sum_{i\ne {i}^{^{\prime}}}^{I}{\left[{y}_{ij}-{y}_{{i}^{^{\prime}}j}-\left({z}_{ij}-{z}_{{i}^{^{\prime}}j}\right)\right]}^{2}}{\left.JI(I-1\right)}.$$

The model producing the smallest MSEP is preferable because it predicts the yield differences in the validation set most accurately.

### Implementation of the models

All models were implemented in SAS 9.4 (SAS Institute 2013) and ASReml-R 4.1.0.130 (Butler et al. 2017). We briefly describe the implementation strategy and visualisations for each package in the Supplementary Material.

### Visualisation

In this section, some visualisation methods are proposed. In SAS, a prediction intervals plot to present predictions with prediction intervals can be generated using PROC SGPLOT. In this plot, the prediction intervals generated based on Eq. 10. A heatmap to present genotype pairwise differences can be generated using PROC TEMPLATE and PROC SGRENDER. The heatmap comprises the *p*-values of the genotype pairwise differences. These p-values are based on the *z*-test. The significance level for the pairwise differences was at *α* = 5%. For Bonferroni adjustment, the significance level α has to be divided by the total number of pairs or the p-values multiplied with the number of pairs (Hochberg and Tamhane 1987). In this case, the *α* was divided with 300 since there were 300 pairs. The *z* score was obtained by division of each predicted pairwise difference by its corresponding SEPD. Another way to present genotype pairwise differences is by generating a table comprising a letter representation of all-pairwise differences can be constructed with the insert-and-absorb algorithm (Piepho 2004). This algorithm is implemented in a SAS macro (Piepho 2012) and can be obtained from https://biostatistik.uni-hohenheim.de/fileadmin/einrichtungen/biostatistik/Tools_und_Macros/SAS-Macros/mult.sas. The basis of significance for this table is the same as the significance in the heatmap. In R, the prediction intervals plot and a heatmap can be generated using the *ggplot2* (Wickham 2016) and *corrplot* (Wei and Simko 2017) packages, respectively.

## Results

The fixed-effects test results of the extended FG1 model for covariate selection are given in Table 3. Based on the F-test, the only significant covariate was the squared scaled clay content. Thus, in this study, we decided to use only the scaled linear and quadratic clay content as covariates for all 14 models.

**Table 3:** Fixed effect tests for covariate selection by the extended FG1 model

Effect*	Numerator DF	Denominator DF	*F* Value	Pr > F
G	24	299	8.91	< .0001
Z	2	5.29	0.18	0.8394
G × Z	48	268	1.27	0.1212
^†^Clay	1	7.69	3.26	0.1100
^†^Clay squared	1	8.2	6.84	0.0303
^‡^pH	1	5.35	2.57	0.1656
^‡^pH squared	1	3.39	0.07	0.8094
^‡^Humus	1	6.87	0.01	0.9312
^‡^Humus squared	1	7.52	0.00	0.9993

^*^G, genotype; Z, zone; G × Z, genotype × zone

^†^Clay content was scaled to $(\mathrm{clay}-40)/10$

^‡^pH and humus were scaled to mean 0 and standard deviation 1

In this section, we present the results of all models fitted by SAS. The results from ASReml-R are available as the Supplementary Materials. The fit statistics report including the deviance, information criterion, ∆Deviance and ∆AIC of the seven FG models and the seven RG models are given in Tables S8 and S9, respectively, in the Supplementary Materials. The fit statistics from ASReml-R are given in Tables S3 and S4 for the seven FG models and seven RG models, respectively.

The covariance parameter estimates of the seven FG models are presented in Table 4. The corresponding ASReml-R outputs are presented in Table S1. The variance estimates for location and genotype × location effects in each zone were highly heterogeneous, as shown for the FG1 model. Compared to the FG2 model, the homogeneous variance structure for the location effect seems not appropriate because the homogeneous variance estimate for location was far larger than the heterogeneous variance estimate for location in the North and South zones, and far smaller for location in the Middle zone. For the genotype × location effect, the homogeneous variance estimate was larger than the heterogeneous genotype × location variance estimate for the North zone, but smaller than for the Middle and South zones. Thus, again, the FG2 model might be less appropriate than the FG1 model. The use of both a linear and a quadratic covariate term (the FGCQ model) decreased the estimate of the inter-location variance compared to the use of only a linear term (the FGC model). However, the estimate of the genotype × location variance did not change much compared to the estimate of the inter-location variance. When the covariates interacted with genotype and genotype × zone, as in the FGI1 model, the estimates of the inter-location variance were larger than the FGCQ model, but the estimate of the variance for the South zone in the genotype × location dropped considerably. There was no difference in any variance component estimates in the FGI1 and FGI2 models. Thus, unexpectedly, when the model comprised fixed effects of genotype × zone and covariates interactions, dropping the covariate interactions with the genotype main effect, variance component estimates did not change. In the FGI3 model, only the genotype main effect interacted with the covariates. Here, the heterogeneous variance component estimates for location and genotype × location effects were similar to the estimates in the FGCQ model.

**Table 4:** Covariance parameter estimates of seven fixed-genotype-effect (FG) models

Covariance	Subject^†^	Group	Estimate*						
parameter			FG1	FG2	FGC	FGCQ	FCI1	FCI2	FCI3
Intercept	L	North	24,606.00		29,725.00	8596.23	11,052.00	11,052.00	8595.45
		Middle	82,207.00		33,466.00	30,888.00	38,357.00	38,357.00	30,893.00
		South	16,142.00		17,068.00	5625.77	6414.33	6414.33	5710.33
				45,118.00					
Intercept	G × L	North	598.57		598.60	598.59	206.18	206.18	534.64
		Middle	1182.36		1182.38	1182.38	1173.21	1173.21	1148.01
		South	1032.08		1032.28	1033.13	117.76	117.76	265.19
				952.72					

*Covariance parameters were estimated by REML

^†^L, location; G × L, genotype × location

Table 5 presents the covariance parameter estimates of the seven RG models. The ASReml-R results for these models are given in Table S2. Unlike the FG models, the RG models included genotype and genotype × zone variance components. The pattern of estimates for inter-location and genotype × location variances were similar to the FG1, FG2, FGC, and FGCQ models. Interestingly, for the RC1, RC2, and RC3 models, the estimate of the genotype × location variance for the South zone dropped substantially compared to the other RG models. However, the estimate of the inter-location variance for the North zone was higher for the RC3 model than for the other RC models.

The average SEPV and SEPD, over 25 genotypes by location and by all 14 models, are presented in Tables 6 and 7, respectively. The results from ASReml-R are given in Table S5 and S6, respectively. The highest SEPV were observed using the RG2 and FG2 models. These two models had no covariate and used a homogeneous variance structure for the location and genotype × location terms. The RC2 and RC3 models were the two models with the smallest SEPV. The RC2 model had the smallest SEPV in the locations N01, N02, and S01, while the RC3 model had the smallest SEPV only in the location S02. However, the difference between the RC2 and the RC3 models for location S02 was subtle. The FGI3 model, with covariate interaction only with the genotype main effect, also had a small SEPV compared with other FG models and even compared with the RG models without any covariate terms. It is also apparent that modelling a heterogeneous variance structure for location and genotype × location terms improved the SEPV compared to the homogeneous variance structure because the RG and FG models had smaller SEPV than the RG2 and FG2. Compared to the FG and RG model, the SEPV in the RC2 model was improved by 37–38% for the untested locations in the South zone, and by 30–35% for the untested locations in the North zone.

**Table 5:** Covariance parameter estimates of seven random-genotype-effect (RG) models

Covariance parameter	Subject^†^	Group	Estimate*						
			RG1	RG2	RGC	RGCQ	RC1	RC2	RC3
Intercept	L	North	24,613.00		29,729.00	8598.12	8608.21	8601.49	8601.40
		Middle	82,195.00		33,452.00	30,874.00	30,887.00	30,872.00	30,817.00
		South	16,120.00		17,048.00	5618.09	5717.27	5715.54	5704.45
		–	–	45,148.00	–	–	–	–	–
Intercept	G		613.46	638.70	613.48	613.55	–	609.96	-
Intercept	G × Z		65.30	60.61	65.28	65.21	–	–	98.88
Intercept	G × L	North	584.45		584.48	584.48	261.99	257.87	551.31
		Middle	1157.63		1157.64	1157.61	1058.87	1060.31	1134.55
		South	1057.85		1058.05	1058.91	309.83	299.28	321.24
		–	–	941.55	–	–	–	–	–
Intercept (1)	G	–	–	–	–	–	460.48	–	527.32
Covariance (2,1)	G	–	–	–	–	–	−121.12	–	−46.90
Linear term slope (2)	G	–	–	–	–	–	61.25	–	415.82
Covariance (3,1)	G	–	–	–	–	–	−9.45	–	−0.43
Covariance (3,2)	G	–	–	–	–	–	30.38	–	207.89
Quadratic term slope (3)	G	–	–	–	–	–	2.53	–	111.97
Intercept (1)	G × Z	–	–	–	–	–	216.39	175.72	–
Covariance (2,1)	G × Z	–	–	–	–	–	113.50	102.30	–
Linear term slope (2)	G × Z	–	–	–	–	–	363.11	453.44	–
Covariance (3,1)	G × Z	–	–	–	–	–	−39.53	−19.84	–
Covariance (3,2)	G × Z	–	–	–	–	–	123.37	169.48	–
Quadratic term slope (3)	G × Z	–	–	–	–	–	98.35	107.75	–

*Covariance parameters were estimated by REML

^†^G, genotype; L, location; G × L, genotype × location; G × Z, genotype × zone

**Table 6:** Averages of SEPV over 25 genotypes of each new location of all 14 models

SEPV	Model	Location			
		N01	N02	S01	S02
		–––––––––– g × m^−2^ ––––––––––			
	RC2	113.13	121.42	88.10	89.38
	RC3	114.23	122.10	88.14	89.20
	RC1	113.18	121.46	88.16	89.45
	FGI3	114.98	122.89	88.19	89.20
	RGCQ	114.24	122.06	91.65	92.57
	FGCQ	114.73	122.52	92.51	93.42
	FGI1	127.76	142.03	92.88	97.02
	FGI2	127.76	142.03	92.88	97.02
	RG1	174.11	174.11	141.55	141.55
	FG1	174.42	174.42	142.17	142.17
	RGC	207.01	215.71	150.87	147.69
	FGC	207.27	215.96	151.45	148.28
	RG2	235.22	235.22	231.91	231.91
	FG2	235.50	235.50	232.21	232.21

**Table 7:** Averages of SEPD over 25 genotypes of each new location of all 14 models

SEPD	Model	Location			
		N01	N02	S01	S02
		–––––––––– g × m^−2^ ––––––––––			
	RC2	29.49	32.54	28.92	30.98
	RC1	29.52	32.50	29.22	31.26
	FGI1	31.36	37.77	24.74	33.16
	FGI2	31.36	37.77	24.74	33.16
	RC3	36.85	37.03	29.61	30.30
	RG1	36.97	36.97	48.39	48.39
	RGCQ	36.97	36.97	48.41	48.41
	RGC	36.97	36.97	48.39	48.39
	FG1	40.03	40.03	51.53	51.53
	FGCQ	40.03	40.03	51.55	51.55
	FGC	40.03	40.03	51.53	51.53
	FGI3	41.62	42.37	29.79	30.16
	RG2	45.84	45.84	45.85	45.85
	FG2	49.55	49.55	49.66	49.66

The SEPD values were smaller than the SEPV values because the computation in Eq. 9 was only based on the terms involving genotype effects. In general, the RC2 model had the smallest SEPD except in location N02. However, again, the difference in SEPD between models RC1 and RC2 was small at this location. The FGI1 and FGI2 model had the same and the smallest SEPD among the FG models. In the FGI1 model, both genotype and genotype × zone terms had interactions with the covariates, while in the FGI2 model, only the genotype × zone term interacted with the covariate.

The heterogeneous variance structure for location and genotype × location was beneficial since the SEPD in the FG1 and RG1 models were smaller than in the FG2 and RG2 models. The SEPD depended on the covariate as well. For the untested locations in the South zone, the SEPD were smaller in the model where the covariate interacted with genotype and genotype × zone terms. Concomitantly, when the covariate had no interactions with genotype and genotype × zone terms, the SEPD of the untested locations in the South zone were higher than in the North zone. The SEPD in the RC2 model decreased by 36–40% for the untested locations in the South zone and by 12–20% for the untested locations in the North zone, which implies that the genotype × location variance estimate in the North zone is much higher than in the South zone. This is also shown in Table 5.

Table 8 presents variance and correlations estimates of the RC2 model, since this was a promising model based on the averages of SEPV and SEPD. The correlation between the linear slope term and the intercept was 0.36, which is low. However, the correlation between the quadratic term and the intercept was -0.14, which is very low as well. The correlation between quadratic slope and the linear slope was quite high, i.e. 0.76. Thus, there was no problem with collinearity between these two terms. The results of ASReml-R are given in Table S7.

**Table 8:** Covariance parameter estimates of RC2 model with correlation between slope and intercept

Covariance parameter	Subject*	Group	Estimate
Intercept	L	North	8601.49
		Middle	30,882.00
		South	5715.53
Intercept	G		609.93
Intercept	G × L	North	257.83
		Middle	1060.30
		South	299.35
Intercept	G × Z		175.42
Linear term slope (2)	G × Z		453.35
Quadratic term slope (3)	G × Z		107.72
Corr(2,1)	G × Z		0.36
Corr(3,1)	G × Z		−0.14
Corr(3,2)	G × Z		0.77

*G, genotype; L, location; G × S, genotype × location; G × Z, genotype × zone

The MSEP of the 14 models are listed in Table 9. The model with the smallest MSEP predicted yield differences in the validation set most accurately. The RG models outperformed the FG models because their MSEP were smaller than the FG models. Hence, although both the FGI1 and FGI2 models had the exact same SEPD, and their SEPD were fairly competitive to the SEPD in the RC models, these two models were the least performant since their MSEP were the highest. The RG1, RGC, and RGCQ models had the smallest MSEP, and their values were the same when they were rounded. The RC model with the random coefficients for the genotype × zone term (RC2) ranked fifth but considering that the three smallest MSEP were equal, the MSEP of the RC2 model can be considered as the third smallest. Clearly, the pattern shows that the models with covariate interaction in the genotype or genotype × zone terms were less performant than the models without such interactions. Based on the MSEP, the best models were RGCQ, RGC, RG1, RG2, and RC2.

**Table 9:** The means squared error of prediction differences of 14 models

Ranking	Model	MSEP (g^2^ × m^−4^)
1	RGCQ	3681
2	RG1	3681
3	RGC	3681
4	RG2	3688
5	RC2	3797
6	RC3	3839
7	RC1	3848
8	FG2	4000
9	FGCQ	4008
10	FG1	4008
11	FGC	4008
12	FGI3	4393
13	FGI1	7217
14	FGI2	7217

**Fig. 1:**
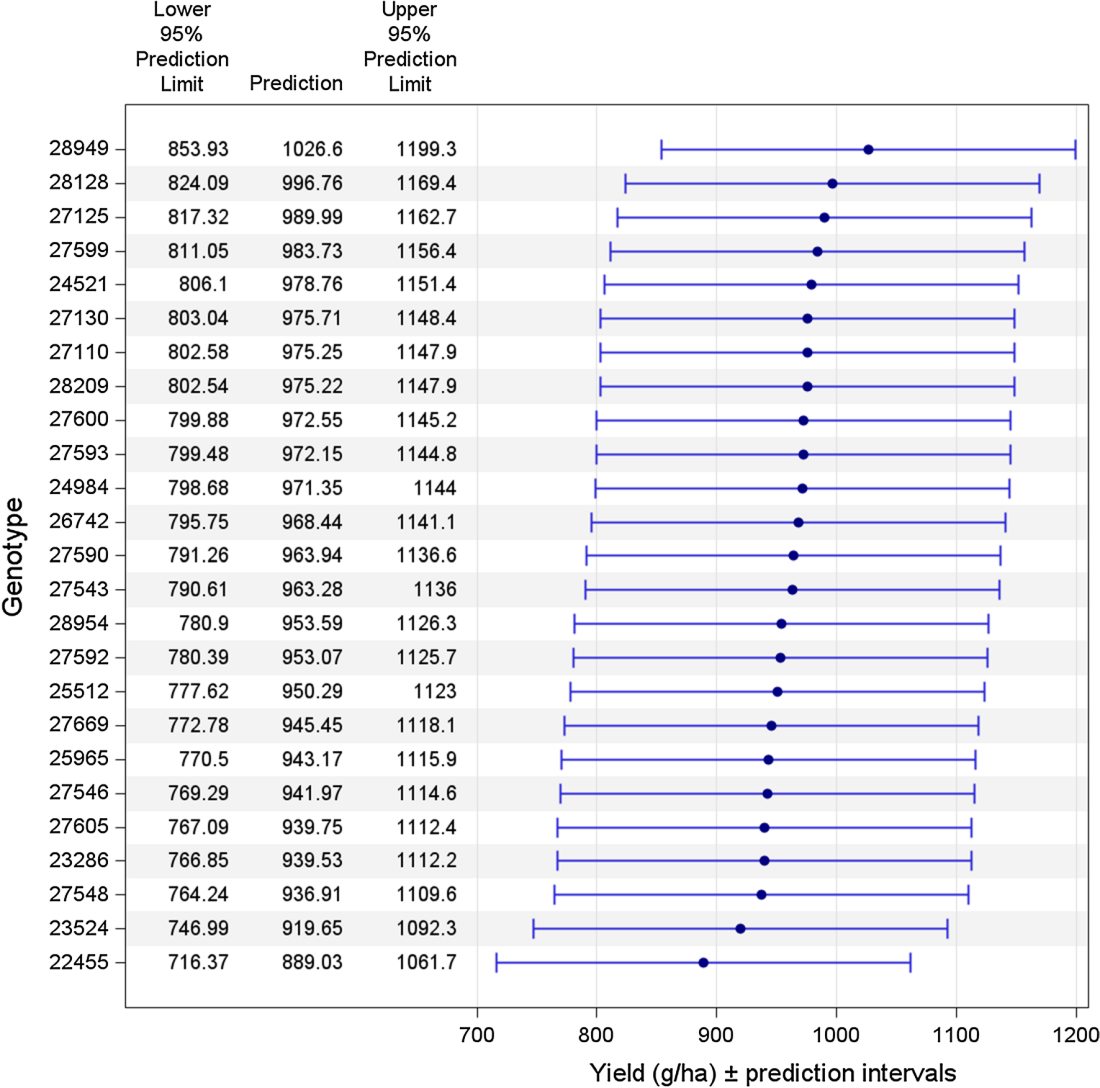
Predictions and 95% prediction intervals for each genotype in location S01 by the RC2 model by PROC SGPLOT

**Fig. 2:**
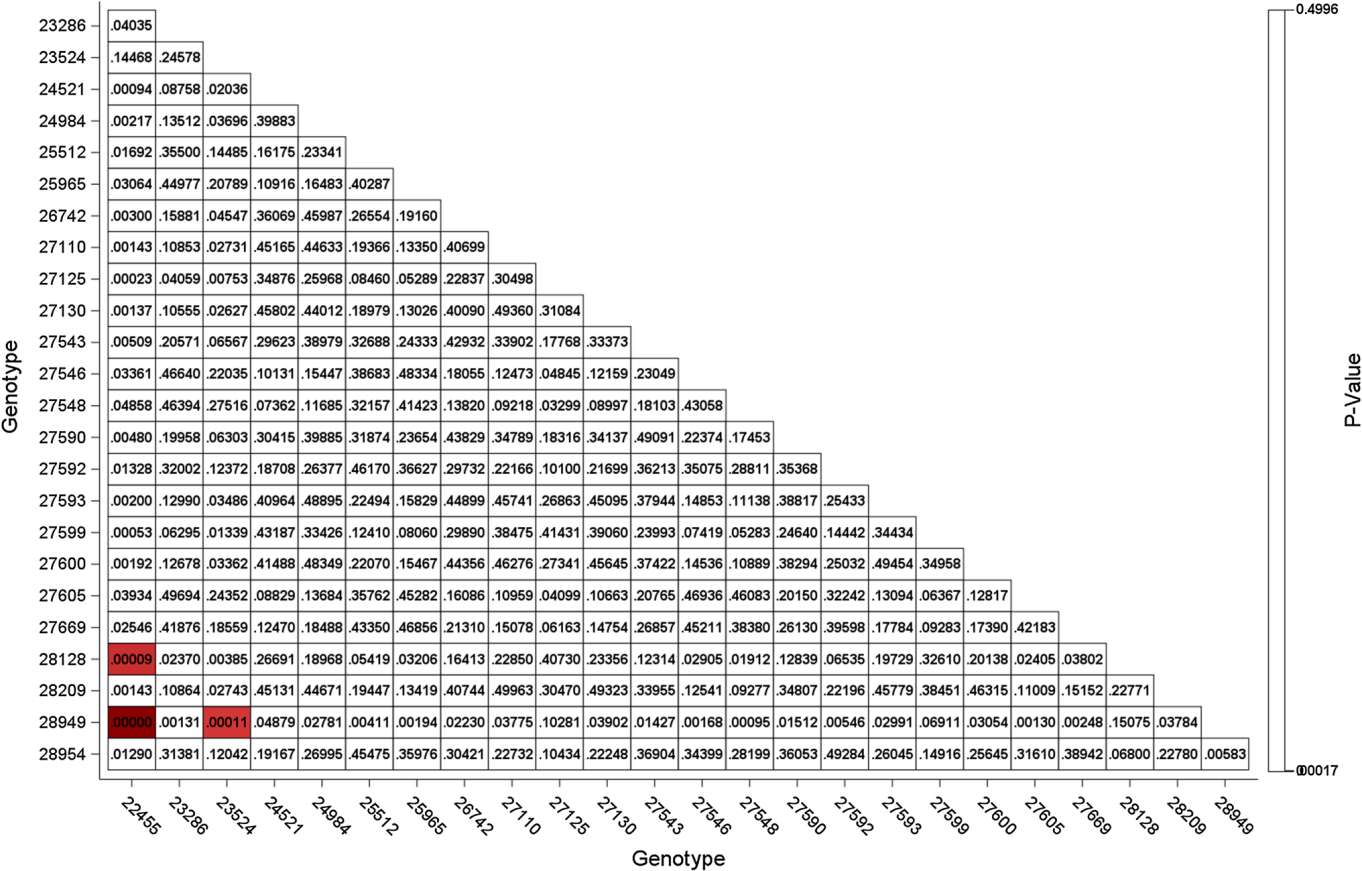
Heatmap of *p*-value of the genotype pairwise differences in location S01 by the RC2 model by PROC TEMPLATE and PROC SGRENDER. The significance level was adjusted using Bonferroni adjustment

We demonstrate how the proposed visualisations of the results can be implemented in SAS and R. Predicted values of the genotypes and the 95% prediction intervals for location S01 based on the RC2 model are depicted in Fig. 1. Figure from R is given in Figure S4. Figure 1 presents the upper and lower prediction limits, which were obtained from Eq. 10. For the prediction of pairwise differences of genotypes in location S01 by the RC2 model, a heatmap containing p-values is given in Fig. 2. The heatmap produced by R is presented in Figure S5. The heatmap can be useful for data with a large number of genotypes and can provide an easy way to detect, by colour coding, which pairwise comparisons of genotypes are significant. The red shading indicates significant differences of pairwise genotypes predictions at *α* = 5% with Bonferroni adjustment. The SAS and R codes for this purpose are available in the electronic Supplementary Material.

The pairwise differences displayed by a letter-based representation using the insert-and-absorb algorithm (Piepho 2004) are given in Table 10. The drawback of presenting letter-based representation of multiple comparisons is the limitation of the alphabet numbers and the clutter when many letters are required. When the needed number of letters exceeds 26, then this method will not work with the Latin alphabet, which can occur for a very large number of genotypes. The SAS code to generate Table 10 in the electronic Supplementary Materials.

**Table 10:** The letter representation of all-pairwise differences by the insert-and-absorb algorithm (Piepho 2004)

Genotype	Yield (g/ha)*	Letters		
28,949	1026.60	a		
28,128	996.76	a	b	
27,125	989.99	a		c
27,599	983.73	a		c
24,521	978.76	a		c
27,130	975.71	a		c
27,110	975.25	a		c
28,209	975.22	a		c
27,600	972.55	a		c
27,593	972.15	a		c
24,984	971.35	a		c
26,742	968.44	a		c
27,590	963.94	a		c
27,543	963.28	a		c
28,954	953.59	a		c
27,592	953.07	a		c
25,512	950.29	a		c
27,669	945.45	a		c
25,965	943.17	a		c
27,546	941.97	a		c
27,605	939.75	a		c
23,286	939.53	a		c
27,548	936.91	a		c
23,524	919.65		b	c
22,455	889.03			c

*Means not sharing any letter are significantly different at the 5% level of significance with Bonferroni adjustment (Piepho, 2018)

## Discussion

In this study, it is shown that using clay in the quadratic term as a covariate and employing RC models reduced the SEPV averages for all new locations by 30–38%, and the SEPD averages by 12–40%. This shows that the RC models can improve the precision of predictions of genotypes performance and the precision of genotypes comparisons. By using the covariates in the random coefficients term, the SEPV is evaluated at specific values of the covariates (Milliken and Johnson 2002), which can substantially decrease the SEPV of the RC models compared to the models without any random coefficients. Between the 14 models, RC2 was the model with the smallest average SEPV for three new locations, except in location S02, the SEPV of the RC2 model was 0.18 higher than the RC3 model. Thus, it was minor. For the average SEPD, the RC2 model had only a marginally larger average SEPD in location N02 than the RC1 model.

Nevertheless, the difference in average SEPD between the RC1 and RC2 models was small. The RC2 model utilised the linear and quadratic term in the genotype × zone interaction effects, but not in the genotype main effects. Figure S1 presents the quadratic regression per genotype and shows that the variation between genotypes is not that large, as the lines of genotypes are close to each other.

For this reason, the inclusion of random coefficients in the genotype term may not be worthwhile. The random coefficients in the genotype × zone term were more beneficial than in the genotype main-effect term, since the SEPD was higher when using the RC3 model than when using the RC2 model. Also, Figure S2 shows that the variation between genotypes × zone effects is large, as the lines of genotypes × zone are more widely spread out compared to Figure S1.

The model selection can be made by jointly considering SEPV and SEPD with MSEP from the CV study. In terms of MSEP, the FG models are clearly not favourable since their MSEP were higher than those of the RG models. This poor performance is expected because in difference to the RG models, the FG models cannot borrow strength across zones. Among the RG models, there was no clear winner when jointly considering the SEPV, SEPD, and MSEP. The precision was certainly improved via the RC2 model, but its MSEP was not the smallest. The models with the smallest MSEP were RGCQ, RGC, and RG. Nevertheless, the difference of the MSEP between the RC2 and the RG, RGC, RGCQ model was 116 g^2^ × m^−4^. Thus, the RC2 model predictions were less accurate $\left(\sqrt{\mathrm{MSEP}}=10.73\mathrm{ g}\times {\mathrm{m}}^{-2}\right)$ compared to those three models. With the RG1 model, the prediction accuracy was slightly better, but the intervals and the uncertainty were larger compared to the RC2 model. Thus, the RC2 model is preferred based on the SEPV, SEPD, and the MSEP. The RC2 model minimised the uncertainty as indicated by lower SEPV and SEPD, although its MSEP was not the smallest.

Our study is similar to Jarquin et al. (2014) in that both use RC models to improve prediction accuracy. The major difference is that Jarquin et al. (2014) used an extensive number of environmental covariates with marker data and utilised this information by computing an environmental kinship matrix, for which a single variance component was fitted. Thus, implicitly, the model used by Jarquin et al. (2014) assumes that the slopes for the different covariates have the same variance and that there is no correlation between them. By contrast, in our study, a vast number of covariates and marker data were not available, but we allowed for heterogeneity in variance between slopes, and for covariance between slopes and intercepts to maintain the invariance feature of RC models (Longford 1993; Piepho and Ogutu 2002; Wolfinger 1996). It is acknowledged that we can afford to do so because we do not have a vast number of covariates. With an increasing number of covariates, fitting such RC models becomes more challenging, and it is not obvious how this can best be done. One option to circumvent numerical problems is to fit a low-rank approximation to the unstructured variance–covariance matrix for intercepts and slopes, i.e. a factor-analytic model (Jennrich and Schuchter 1986). Fitting a factor-analytic model guarantees that the variance–covariance matrix is positive definite. If the order of the factor-analytic model equals the number of slope terms plus the intercept, the model is equivalent to the unstructured model, whereas lower-rank approximations are obtained by reducing the order. Moreover, with a large number of covariates, covariate selection will be beneficial. R-square (R^2^) for mixed models (Piepho 2019) is an option for covariate selection, as demonstrated in Hadasch et al. (2020). The best approach to accommodating a larger number of covariates certainly deserves further research.

The BLUPs for the untested locations are the predictions obtained from the observed locations, and these values are reported to growers. However, these BLUPs do not equal the yield that the growers will get in practice. Furthermore, the standard errors of the predictions based on the observed locations cannot be applied to the grower’s fields since the trials hardly ever coincide with these. Hence, the prediction intervals for the untested locations need to be computed and presented so that the growers have a view of how precise each cultivar’s prediction is for their fields. To compute valid standard errors for the untested locations, the location effect needs to be modelled as random to account for the uncertainty of effects for the untested locations. Note that the random location main effect, as well as the random cultivar-location interaction effects, are part of the deviations from the regression curves. Prediction intervals are passed on the random variation around the curves. Hence, only if these effects are indeed present and modelled as random, can we compute valid prediction intervals for new locations. If the location effect is fixed, which is known as factorial regression, then the untested locations’ uncertainty cannot be obtained since there is no variance component estimate for a fixed effect and the effect itself is unobservable. Besides, when the location effect is fixed, the standard errors are only valid for the locations where the trials are carried out. The next step, after having developed the uncertainty measure of the untested locations, could be to apply the RC model for a whole country to present maps of yield predictions using environmental covariates based on the available GIS data in the Swedish official cultivar trials.

When the covariate was modelled using the quadratic term, the averages of SEPV dropped considerably. Furthermore, using RC models, the reduction of the averages SEPV and SEPD depends on the covariate value in the new locations; Table 8 shows that the reduction of SEPV and SEPD varied between the four new locations. Gozdowski et al. (2017) reported that the relationships between the fine soil fractions for the 0–60 cm layers and yield are curvilinear, which also was the case in this study. The clay content used in this study was measured for the 0–25 cm layer. The SEPV depends on the variance estimates of location and genotype × location. The SEPV will decrease when the variance of location or genotype × location decrease. For the SEPD, however, using a covariate did not decrease SEPD because the SEPD only depends on the terms associated with genotype. Moreover, all variance estimates of effects for genotype, genotype × zone, and genotype × location, are similar for the RG1, RG2, RGC, and RGCQ models, while for the RC models these three terms have different variance estimates compared to the RG1, RG2, RGC, and RGCQ models (Table 5). For the FG models, the same pattern occurred in that the genotype × location variance estimates of FG1, FG2, FGC, and FGCQ models were similar.

The covariate scale is crucial when implementing RC models. We found this covariate scale issue when we fitted the RC1 model. This scaling issue occurred when the random coefficients were fitted to both genotype and genotype × zone effects:

1. When the value subtracted from the covariate was lower than the covariate’s mean, and the result was divided by 10, the variance component of the linear term of the random coefficients of the genotype main effect was 0.
2. The same results were obtained when the covariate was standardised to mean 0 and standard deviation 1 and when the value subtracted from the covariate was the covariate’s mean, and the result was divided by 10.

The genotype main effect had a lower variance than the genotype × zone interaction effect, as shown in Figures S1 and S2. Thus, there was likely competition between genotype main effects and genotype × zone interaction effects in absorbing the variance because in the RC2 model, in which the random coefficients were only fitted for the genotype × zone interaction effect, the scaling of (1) and (2) caused no issue in the variance component estimates and the convergence. Besides, using the scaling of (1) and (2) in the RC2 model did not change the SEPV and SEPD for all new locations due to the invariance property ensured by allowing a covariance among random coefficients (unstructured variance–covariance). Other scalings such as subtraction-of-the-minimum and covariate-centring were also attempted. When these scalings were used, the RC models did not converge. Thus, the covariate scaling is essential for model convergence and to obtain the appropriate variance parameter estimates. The clay covariate was scaled for all fitted models by $(\mathrm{clay}-40)/10$. We used this scaling because it yielded a positive definite variance–covariance matrix. The choice of covariates is important in the fixed-effects and random-coefficients parts since they contribute to the improvement or worsening of the prediction precision. Furthermore, the scaling and linear transformations of the covariate are also essential since it can affect convergence and the estimates of covariance parameters.

Zone stratification improved precision because information can be borrowed between zones. However, it should be emphasized that a clear agro-ecological division is needed in order to improve precision for each zone. In this study, the North zone had slightly higher uncertainty than the South zone, which shows that in the North zone, geographical and environmental conditions might be more varied and predictions more uncertain. The SEPV of a zone can be improved by conducting more trials within a zone.

The RC models are useful for MET analysis because the covariate information, which is changing from one to the other trials, can be exploited in the random effects. This feature is useful to measure the adaptability of the genotypes in new environments, given the covariates of the new environment are available. In fact, the benefit of using a covariate in the RC models depends on choosing the appropriate covariate. Covariates can initially be selected based on the biological considerations. Still, it is necessary to check whether these covariate candidates improve the model fit.

The computational approach for predictions in the new locations is different from Henderson (1977), who considered predictions for animals that were not in the records. The main difference is that in our models the location-specific random effects are independent between the observed dataset and the new location, whereas with the Henderson (1977) approach the random effects for animals with records are correlated by pedigree with the animals having no records. Moreover, if there is no zone stratification, the predictions of genotypes in the new locations are merely the summation of genotype BLUPs and the grand mean.

In our study, the precision of these predictions was additionally improved by incorporating covariates using RC models in terms of decreasing standard errors of predictions and standard error of pairwise differences of genotype predictions. Using RC models requires that covariates are available at each location. When the dataset is augmented with data of the new location, the computing strategy needs to be adjusted due to fact that there are no observations on the response in the new locations. For SAS, the augmented dataset could be used to conduct the analysis. However, for ASReml-R, the augmented dataset could not be used due to a different functionality of the package. Thus, the computing strategy needed to be adjusted by excluding the new locations from the dataset to conduct the analysis. The SEPV and SEPD of the new locations had to be computed after the analysis was completed in ASReml-R (see Supplementary Materials).

We demonstrated that predicting genotype yield for some new locations can be enhanced by inclusion of environmental covariates by borrowing information from other zones. A recent paper by Neyhart et al. (2021) followed the same spirit of predicting genotype performance in unobserved environments. Neyhart et al. (2021) assessed the genome-wide predictions in the unobserved environments for both between and within breeding generations. Resende et al. (2020) recently proposed the geospatial (geographic information system) genotype–environment interaction (GIS–GEI) method within an enviromics framework. This framework involves the joint analysis of MET data accounting for phenotypic, genotypic and envirotypic sources of information. It is anchored into a geoprocessing environment that employs enviromic markers, e.g. time-trend climate data, landscape or and management treatment information, obtained by means of modern envirotyping techniques. Our proposed RC modelling approach is ideally suited for integration in an enviromics-driven GIS–GEI framework. Moreover, the RC modelling can be used in the Bayesian framework as proposed by Theobald et al. (2002), who used a Bayesian method for making predictions with incorporating environmental covariates.

## Conclusion

This study showed that the RC models can be used to improve the precision of the yield predictions of winter wheat genotypes in some new locations. The RC model RC2 was competitive, with regards to MSEP, compared to the RG, RGC, and RGCQ models. The RC2 model, with random coefficients of linear and quadratic terms in the genotype × zone effect, can be recommended based on joint consideration of precision in predictions and accuracy. The RC models improved the precision of the predictions for a new location by utilising covariate information in the new location in the random effects part, and by borrowing information from other zones via genetic correlation between zones. The crucial keys for improving the precision of the predictions in the RC models are the selection of suitable covariates, suitably scaling the covariate, modelling the appropriate trend for the covariate in the fixed-effects part, and using an unstructured variance–covariance for the random coefficients. The scale of the covariate is essential to obtain reliable variance component estimates and avoid convergence issues. Failing to select suitable covariates and to model the trend for the covariate also in the fixed-effects part may lead to larger SEPV and SEPD for new locations. Breeders can use RC models to determine the adaptability of tested genotypes in new environments. Agronomists and growers can use RC models to identify the best locally adapted genotypes.

### Supplementary information

Supplementary file1 (ZIP 1921kb)

## Data Availability

The SAS and R code are available as electronic Supplementary Materials.

## Appendix

The differences between three types of inference space in McLean et al. (1991) are explained as follows:

1. The broad inference space refers to inference based exclusively on fixed effects and estimable functions, ${\mathbf{K}}^{\mathbf{^{\prime}}}{\varvec{\upbeta}}$. For example, the estimable function for the adjusted mean of the Middle zone is obtained by choosing $\mathbf{K}\mathbf{^{\prime}}$ accordingly, i.e. ${\mathbf{K}}^{\mathbf{^{\prime}}}=[\mathrm{1,1},0,\dots ,0]$ gives ${\mathbf{K}}^{\mathbf{^{\prime}}}{\varvec{\upbeta}}=\mu +{\zeta }_{1}$, where the first coefficient of 1 refers to the grand mean, $\mu$, and the second to the fixed effect of the Middle zone, ${\zeta }_{1}$. This is so, because the levels of the zone effect are ordered alphabetically such that the effect of the Middle zone comes first.
2. The narrow inference space refers to predictable functions, but is not subject-specific. In the example for the broad inference space, the average of the Middle zone is determined by $\mu +{\zeta }_{1}$. There are no random effects involved. Alternatively, we may consider the average of the Middle zone conditionally on the random genotype effects, which corresponds to the narrow inference space. The predictable function then is $\mu +{\zeta }_{1}+{\bar{g}}+{\left({{\overline{{g}\zeta} }}\right)}_{1}$, where ${\bar{g}}$ is the average of the main random genotype effect, ${I}^{-1}\sum {g}_{i}$, and ${\left({{\overline{{g}\zeta }}}\right)}_{1}$ is the genotype average in the random genotype × zone interaction effect in the Middle zone, ${I}^{-1}\sum \sum {\left({\zeta{g} }\right)}_{i1}$.
3. The intermediate inference space also involves random effects, but in difference to the narrow inference space, the intermediate inference space is subject-specific. For example, the performance of *i*-th genotype in the *j*-th location of the Middle zone is given by $\mu +{\zeta }_{1}+{s}_{j1}+{g}_{i}+{(g\zeta )}_{i1}+(g{s)}_{ij1}$. The estimable function $\mathbf{K}\mathbf{^{\prime}}{\varvec{\upbeta}}$ is $\mu +{\zeta }_{1}$ and the predictable function $\mathbf{M}\mathbf{^{\prime}}\mathbf{u}$ is ${s}_{j1}+{g}_{i}+{(g\zeta )}_{i1}+(g{s)}_{ij1}$.

## Data availability

The data used in this study are available as electronic Supplementary Materials.
