# Supplementary material for: Projecting results of zoned multi-environment trials to new locations using environmental covariates with random coefficient models: accuracy and precision
Source: Theor Appl Genet. 2021 Apr 8;134(5):1513–30. doi: 10.1007/s00122-021-03786-2 (PMC8081717; doi:10.1007/s00122-021-03786-2)
Supplement: Supplementary file 1 — Supplementary file1 (ZIP 1921kb) [file 122_2021_3786_MOESM1_ESM.zip › 122_2021_3786_MOESM1_ESM/Fit statistics from SAS.pdf]

Fit statistics from SAS

The deviance, which is presented as minus 2 times the log-likelihood, and the Akaike information criterion (AIC) were obtained by using the maximum likelihood (ML) method because these models differed in the fixed effects and random effects (Wolfinger 1993).

Table S8. Deviance, Akaike information criterion (AIC), ΔDeviance, and ΔAIC of the seven fixed-genotype-effect (FG) models.

**Model**	**Deviance†**	**AIC†**	**ΔDeviance**	**ΔAIC**
FG1*	4622.1	4784.1	-	-
FG2	4634.9	4788.9	12.8	4.8
FGC	4613.0	4781.0	-9.1	-3.1
FGCQ	4598.3	4768.3	-23.8	-15.8
FGI1	4214.9	4672.9	-407.2	-111.2
FGI2	4214.9	4672.9	-407.2	-111.2
FGI3	4432.3	4696.3	-189.8	-87.8

*The FG1 model is the reference model.
†Obtained via maximum likelihood.

Table S9. Deviance, Akaike information criterion (AIC), ΔDeviance, and ΔAIC of the seven random-genotype-effect (RG) models.

**Model**	**Deviance†**	**AIC†**	**ΔDeviance**	**ΔAIC**
RG1*	4766.6	4788.6	-	-
RG2	4777.5	4791.5	10.9	2.9
RGC	4757.4	4785.4	-9.2	-3.2
RGCQ	4742.8	4772.8	-23.8	-15.8
RC1	4698.4	4748.4	-68.2	-40.2
RC2	4701.1	4741.1	-65.5	-47.5
RC3	4712.9	4752.9	-53.7	-35.7

*The RG1 model is the reference model.
†Obtained via maximum likelihood.
