# Supplementary material for: Projecting results of zoned multi-environment trials to new locations using environmental covariates with random coefficient models: accuracy and precision
Source: Theor Appl Genet. 2021 Apr 8;134(5):1513–30. doi: 10.1007/s00122-021-03786-2 (PMC8081717; doi:10.1007/s00122-021-03786-2)
Supplement: Supplementary file 1 — Supplementary file1 (ZIP 1921kb) [file 122_2021_3786_MOESM1_ESM.zip › 122_2021_3786_MOESM1_ESM/Implementation of the Models in SAS and ASReml.pdf]

Implementation of the Models in SAS and ASReml-R

SAS

All models were fitted using PROC MIXED and PROC GLIMMIX, and the computation of SEPV and SEPD were done in PROC IML in SAS 9.4 (SAS Institute 2013). In stage I, using PROC MIXED, the means μ_{ijm}, were estimated using generalised least squares.

For the new location, in this stage, dummy observation values were put in the **Y** vector as well as the variance-covariance matrix **R**. The two-stage analysis SAS macro from Damesa et al. (2017) was used to construct the **R** matrix and combine it into a single dataset. The number of rows and columns of the matrix **R** had to be the same as the number of records in the dataset, as shown in Figure 1. We denote by $\mathbf{R}^*$ the matrix **R** augmented by a dummy variance-covariance matrix for the new location, $\mathbf{R_0}$. The actual values assigned to elements in $\mathbf{R_0}$ are immaterial, as this matrix is not actually used, but it must be present for the procedure to do the calculations. Figure 1 presents a simple example of a matrix $\mathbf{R}^*$ for a toy dataset consisting of two genotypes in three locations including a new location (S0). The values in the blue shaded part is the matrix $\mathbf{R_0}$. The matrix **R** only includes the variances and covariances of genotypes for the locations that have information (only the green shaded part, comprising matrices $\mathbf{R}_j$, for $j = 1, 2, \ldots$; see Figure S3). Hence, the matrix **R** from stage 1 will not have the same dimension as the matrix $\mathbf{R}^*$, which is augmented with the new locations. The dummy observation was needed to construct the design matrix for stage II analysis. The design matrix, in turn, is needed to compute genotype predictions, prediction of pairwise differences, SEPV, and SEPD using PROC IML. This procedure was used because of the limitation of the ESTIMATE statement of PROC MIXED in handling multiple subjects. In stage II, these dummy values of the new location were removed, and the analyses were carried out using PROC MIXED.

The computation of SEPV and SEPD in PROC IML utilised the design matrix from PROC GLIMMIX and the matrix inverse of left-hand side of MME in Equation 2, denoted as matrix $\hat{\mathbf{C}}$, which is obtained via the MMEQSOL option of PROC MIXED. The last column in the dataset produced by MMEQSOL had to be dropped since the last column comprised the right-hand side of the MME. The SAS codes are available in the electronic Supplementary Materials.

Row	Col1	Col2	Col3	Col4	Col5	Col6	Location	Genotype	Adj.Mean
1	0	0	0	0	0	0	S0	A	.
2	0	0	0	0	0	0	S0	B	.
3	0	0	0.14	0	0	0	S1	A	15.40
4	0	0	0	0.14	0	0	S1	B	17.46
5	0	0	0	0	0.21	0	S2	A	15.80
6	0	0	0	0	0	0.21	S2	B	17.50

Figure S3. A simple illustration of matrix $\mathbf{R}^*$ (Col1 – Col6) with a dataset consisting of two genotypes and three environments including a new environment. The blue shaded part, matrix $\mathbf{R}_0$, cannot be empty but the values are immaterial. The green shaded part, matrix $\mathbf{R}$, must comprise the actual variances and covariances of adjusted genotype means in the locations with data.

ASReml-R

In ASReml-R version 4.1.0.130 (Butler et al. 2017), it was not possible to directly fit all the models using a dataset with the new locations included. Since the new locations had no information, this resulted in no variation. The new locations, therefore, had to be excluded to fit the models. Furthermore, a data frame for each new location had to be generated since it is needed to obtain the EBLUE or EBLUP, SEPV, and SEPD for each new location. Thus, since there were four new locations, four new data frames were generated. After the model was fitted, the `predict.asreml` function was used to obtain the EBLUE or EBLUP of the new locations. In the `predict.asreml` function, the `levels` argument had to be filled with the factor levels based on the new location data frame. The SEPV obtained from this function were the SEPV of either EBLUE or EBLUP at zone level, corresponding to $\sqrt{\mathrm{var}(\eta)}$ in Equation 8. The variance

$\mathrm{var}(w|\boldsymbol{\beta}, \mathbf{u})$ in Equation 8 had to be obtained from the location and genotype×location variance component estimates. Finally, the SEPV for the new location was obtained by taking the square root of the sum of the square of the SEPV of the EBLUE or EBLUP zone level and variance component estimates of location and genotype×location effects.

The `sed` statement in the `predict.asreml` was set to `TRUE` to obtain the SEPD. However, the SEPD directly obtained was not correct for the new location because it was only equal to $\sqrt{VDIFF(\hat{\eta})}$ in Equation 10. Hence, to compute the correct SEPD for the new location, the SEPD of the EBLUP zone level was squared and the variance component estimates of location and genotype×location was incorporated, as shown in Equation 10. For the estimate of the pairwise prediction differences in Equation 9, the *asremlPlus* package (Brien 2020) was used.

For computing the model deviance, as the ML method could not be executed in ASReml-R, instead of minus 2 times the log-likelihood, the deviance was computed with the so-called "full likelihood" via the `icREML` function written by (Verbyla 2019). The *asremlPlus* package also computes the full likelihood via `infoCriteria` function by using the option `IClikelihood="full"`. Thus, the deviance and AIC were computed based on the full likelihood with a single iteration based on the variance component estimates obtained using REML method. The R codes are available in the electronic Supplementary Materials.

References

Brien C (2020) AsremlPlus 4.2–26. Adelaide

Butler DG, Cullis B, Gilmour A, Gogel BJ, Thompson R (2017) ASReml-R reference manual, version 4. University of Wollongong, Wollongong

SAS Institute (2013) SAS for Windows 9.4. SAS Inst., Cary, NC

Verbyla AP (2019) A note on model selection using information criteria for general linear models estimated using REML. Aus N Z J Stat 61:39–50. https://doi.org/10.1111/anzs.12254

Wolinger RD (1993) Covariance structure selection in general mixed models. Commun Stat Simul Comput 22:1079–1106. https://doi.org/10.1080/03610919308813143
