# Supplementary material for: Projecting results of zoned multi-environment trials to new locations using environmental covariates with random coefficient models: accuracy and precision
Source: Theor Appl Genet. 2021 Apr 8;134(5):1513–30. doi: 10.1007/s00122-021-03786-2 (PMC8081717; doi:10.1007/s00122-021-03786-2)
Supplement: Supplementary file 1 — Supplementary file1 (ZIP 1921kb) [file 122_2021_3786_MOESM1_ESM.zip › 122_2021_3786_MOESM1_ESM/Supplementary Materials ASReml-R Results.pdf]

Results from ASReml-R

Table S1. Covariance parameter estimates of seven fixed-genotype-effect (FG) models.

Covariance parameter	Subject[†]	Group	Estimate*						
			FG1	FG2	FGC	FGCQ	FC1	FC2	FC3
Intercept		North	24606.02		29725.26	8596.24	11051.97	11051.97	8595.46
	L	Middle	82252.55		33474.02	30892.85	38420.86	38420.86	30897.48
		South	16141.84		17068.22	5625.76	6414.30	6414.30	5710.31
				45158.66					
Intercept		North	598.61		598.64	598.64	206.20	206.20	534.69
	G×L	Middle	1182.36		1182.38	1182.38	1173.21	1173.21	1148.02
		South	1032.12		1032.33	1033.18	117.79	117.79	265.23
				952.74					

*Covariance parameters were estimated by REML.

[†]L, location; G×L, genotype×location.

Table S2. Covariance parameter estimates of seven random-genotype-effect (RG) models.

Covariance parameter	Subject†	Group	Estimate*						
			RG1	RG2	RGC	RGCQ	RC1‡	RC2	RC3
Intercept	L	North	24612.55		29728.47	8598.13	8608.22	8601.54	8601.45
		Middle	82239.83		33459.21	30878.55	30891.65	30882.02	30881.14
		South	16119.87		17047.56	5618.08	5717.24	5715.55	5704.44
		-	-	45150.88	-	-	-	-	-
Intercept	G		613.46	638.70	613.48	613.55	-	610.14	-
Intercept	G×Z		65.30	60.61	65.27	65.21	-	-	98.86
Intercept	G×L	North	584.45		584.53	584.53	261.97	258.24	551.34
		Middle	1157.63		1157.64	1157.61	1058.93	1060.10	1134.55
		South	1057.89		1058.10	1058.95	309.94	299.11	321.31
		-	-	941.57	-	-	-	-	-
Intercept (1)	G	-	-	-	-	-	460.47	-	527.32
Covariance (2,1)	G	-	-	-	-	-	-121.12	-	-46.88
Linear term slope (2)	G	-	-	-	-	-	61.19	-	415.81
Covariance (3,1)	G	-	-	-	-	-	-9.45	-	-0.43
Covariance (3,2)	G	-	-	-	-	-	30.35	-	207.88
Quadratic term slope (3)	G	-	-	-	-	-	2.51	-	111.97
Intercept (1)	G×Z	-	-	-	-	-	216.33	175.89	-
Covariance (2,1)	G×Z	-	-	-	-	-	113.31	103.02	-
Linear term slope (2)	G×Z	-	-	-	-	-	362.81	454.56	-
Covariance (3,1)	G×Z	-	-	-	-	-	-39.65	-19.45	-
Covariance (3,2)	G×Z	-	-	-	-	-	123.26	169.88	-
Quadratic term slope (3)	G×Z	-	-	-	-	-	98.34	107.82	-

*Covariance parameters were estimated by REML.

†G, genotype; L, location; G×L, genotype×location; G×Z, genotype×zone.

‡Estimated with `uspd` set to false.

In ASReml-R, the RC1 model did not converge properly without allowing non-positive definite variance-covariance matrix. Thus, in ASReml-R, the `uspd` was set to `FALSE`. The `uspd` option was set whether the resulting variance-covariance matrix estimate was positive definite or not. The default setting of the unstructured variance-covariance is always bounded to be positive in ASReml-R.

In ASReml-R, the deviance and AIC were computed using the `icREML` function.

Table S3. Deviance, Akaike information criterion (AIC), ΔDeviance, and ΔAIC of the seven fixed-genotype-effect (FG) models.

Model	Deviance†	AIC†	ΔDeviance	ΔAIC
FG1*	-1901.7	3965.5	-	-
FG2	-1908.1	3970.2	-6.4	4.7
FGC	-1897.7	3963.3	4.0	-2.2
FGCQ	-1890.6	3951.3	11.1	-14.2
FGI1	- Inf‡	Inf‡	-	-
FGI2	- Inf‡	Inf‡	-	-
FGI3	-1818.4	3902.8	83.3	-62.7

*The M0 model is the reference model.
†Obtained via full likelihood.
‡The full likelihood returned inf for the deviance and AIC.

Table S4. Deviance, Akaike information criterion (AIC), ΔDeviance, and ΔAIC of the seven random-genotype-effect (RG) models.

Model	Deviance†	AIC†	ΔDeviance	ΔAIC
RG1*	-1969.9	3961.8	-	-
RG2	-1975.4	3964.7	-5.5	2.9
RGC	-1965.9	3959.7	4.0	-2.1
RGCQ	-1958.8	3947.7	11.1	-14.1
RC1	-1936.6	3923.2	33.3	-38.6
RC2	-1938.0	3915.9	31.9	-45.9
RC3	-1943.9	3927.7	26.0	-34.1

*The M0 model is the reference model.
†Obtained via full likelihood.

Table S5. Averages of SEPV over 25 genotypes of each new location of all 14 models.

Precision measures	Model	Location			
		N01	N02	S01	S02
		-------------------- $g.m^{-2}$ --------------------			
SEPV	RC2	113.13	121.42	88.10	89.38
	RC3	114.23	122.10	88.14	89.20
	RC1	113.18	121.46	88.16	89.45
	FGI3	114.98	122.89	88.19	89.20
	RGCQ	114.24	122.07	91.65	92.57
	FGCQ	114.73	122.52	92.52	93.42
	FGI1	127.76	142.03	92.88	97.02
	FGI2	127.76	142.03	92.88	97.02
	RG1	174.11	174.11	141.55	141.55
	FG1	174.42	174.42	142.17	142.17
	RGC	207.01	215.71	150.87	147.69
	FGC	207.27	215.96	151.45	148.28
	RG2	235.23	235.23	231.92	231.92
	FG2	235.61	235.61	232.32	232.32

Table S6. Averages of SEPD over 25 genotypes of each new location of all 14 models.

**Precision measures**	**Model**	**Location**			
		**N01**	**N02**	**S01**	**S02**
		------------------- $g.m^{-2}$ -------------------			
SEPD	RC2	29.50	32.55	28.91	30.98
	RC1	29.52	32.50	29.22	31.26
	FGI1	31.36	37.77	24.74	33.16
	FGI2	31.36	37.77	24.74	33.16
	RC3	36.85	37.03	29.62	30.31
	RG1	36.97	36.97	48.39	48.39
	RGC	36.97	36.97	48.39	48.39
	RGCQ	36.97	36.97	48.41	48.41
	FG1	40.03	40.03	51.53	51.53
	FGC	40.03	40.03	51.53	51.53
	FGCQ	40.03	40.03	51.53	51.53
	FGI3	41.62	42.37	29.79	30.16
	RG2	45.84	45.84	45.86	45.86
	FG2	49.55	49.55	49.66	49.66

Table S7. Covariance parameter estimates of RC2 model with correlation between slope and intercept.

**Covariance parameter**	**Subject***	**Group**	**Estimate**
Intercept	L	North	8601.53
		Middle	30882.00
		South	5715.53
Intercept	G		610.07
Intercept	G×L	North	258.12
		Middle	1060.18
		South	299.20
Intercept	G×Z		175.82
Linear term slope (2)	G×Z		454.10
Quadratic term slope (3)	G×Z		107.79
Corr(2,1)	G×Z		0.36
Corr(3,1)	G×Z		-0.14
Corr(3,2)	G×Z		0.76

*G, genotype; L, location; G×L, genotype×location; G×Z, genotype×zone.

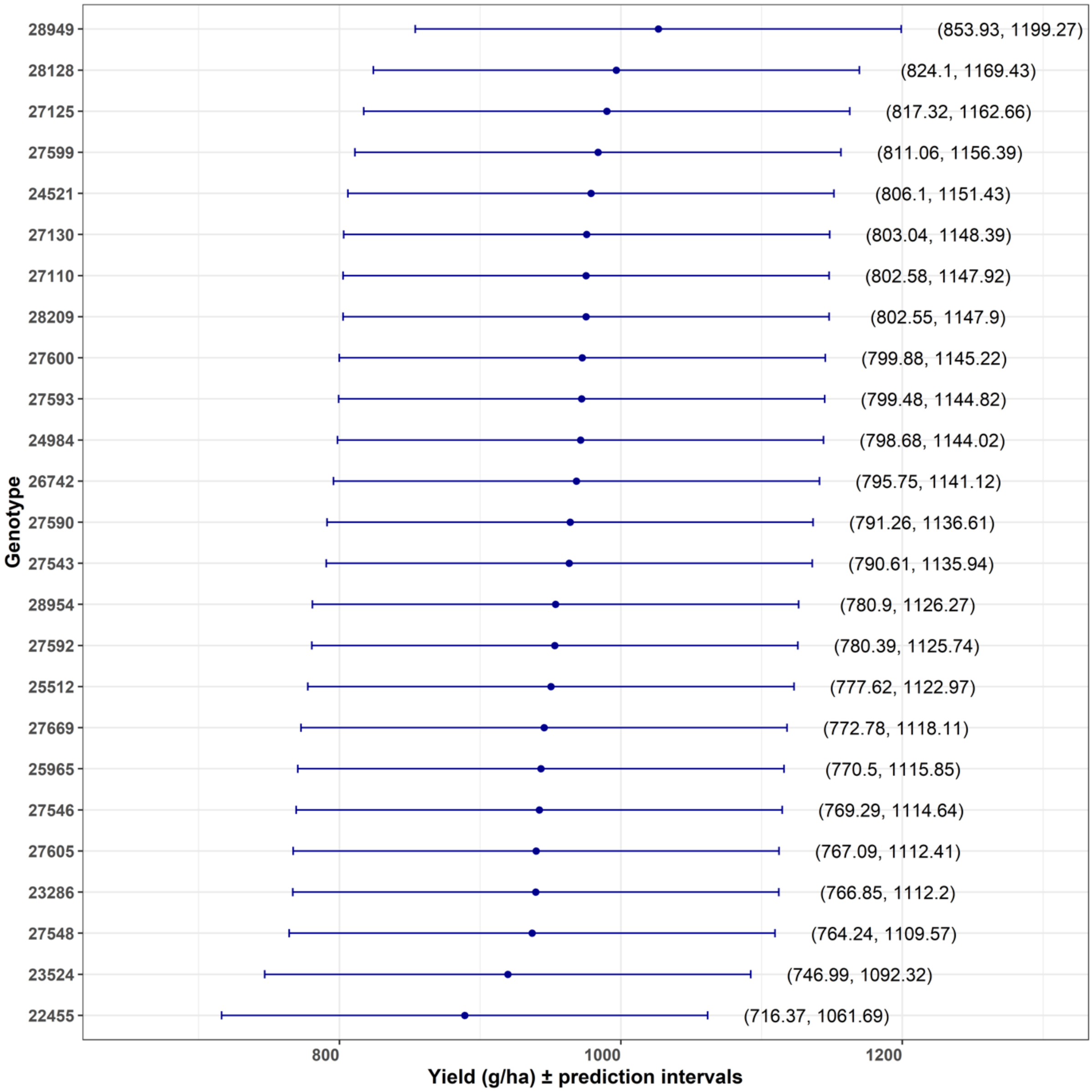

Figure S4. Predictions and 95% prediction intervals for each genotype in location S01 by the RC2 model by *ggplot2* package in R.

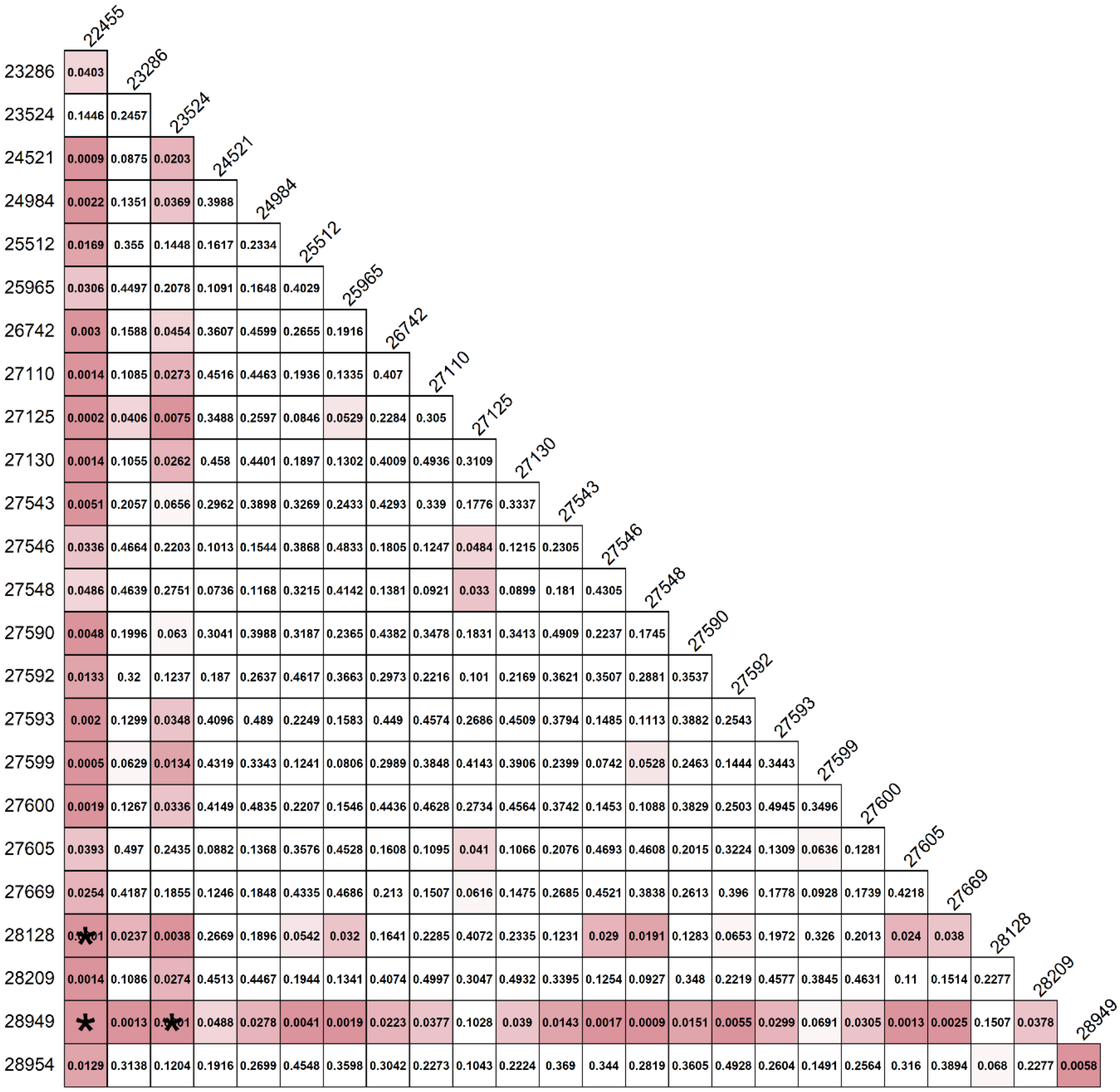

Figure S5. Heatmap of p-values of the genotype pairwise differences in location S01 by the RC2 model by *corrplot* package in R. The asterisk shows the significant difference based on Bonferroni adjustment.
