# Supplementary figures and images for: Projecting results of zoned multi-environment trials to new locations using environmental covariates with random coefficient models: accuracy and precision
Source: Theor Appl Genet. 2021 Apr 8;134(5):1513–30. doi: 10.1007/s00122-021-03786-2 (PMC8081717; doi:10.1007/s00122-021-03786-2)

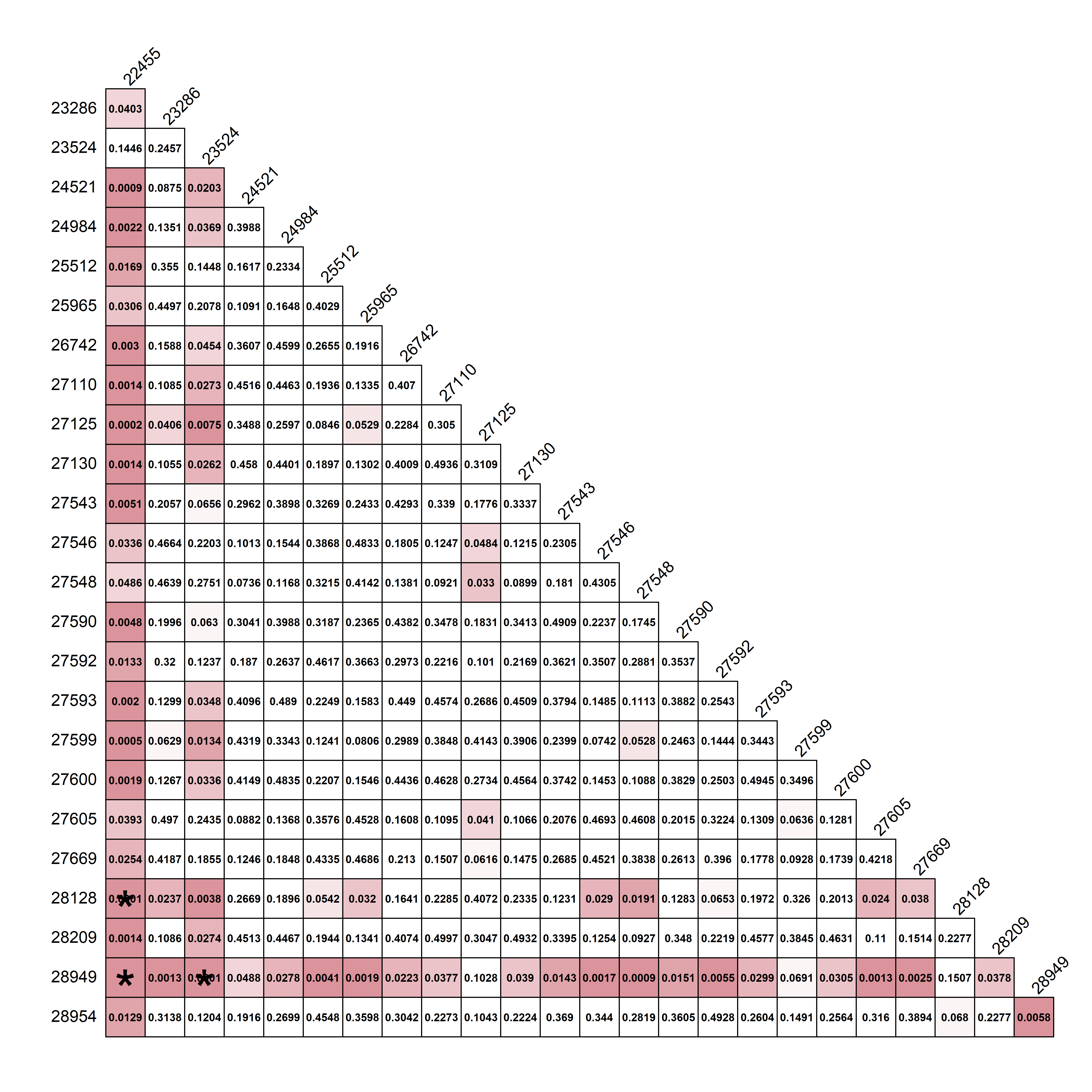

Supplement: Supplementary file 1 — Supplementary file1 (ZIP 1921kb) [file 122_2021_3786_MOESM1_ESM.zip › 122_2021_3786_MOESM1_ESM/Heatmap_R.png]

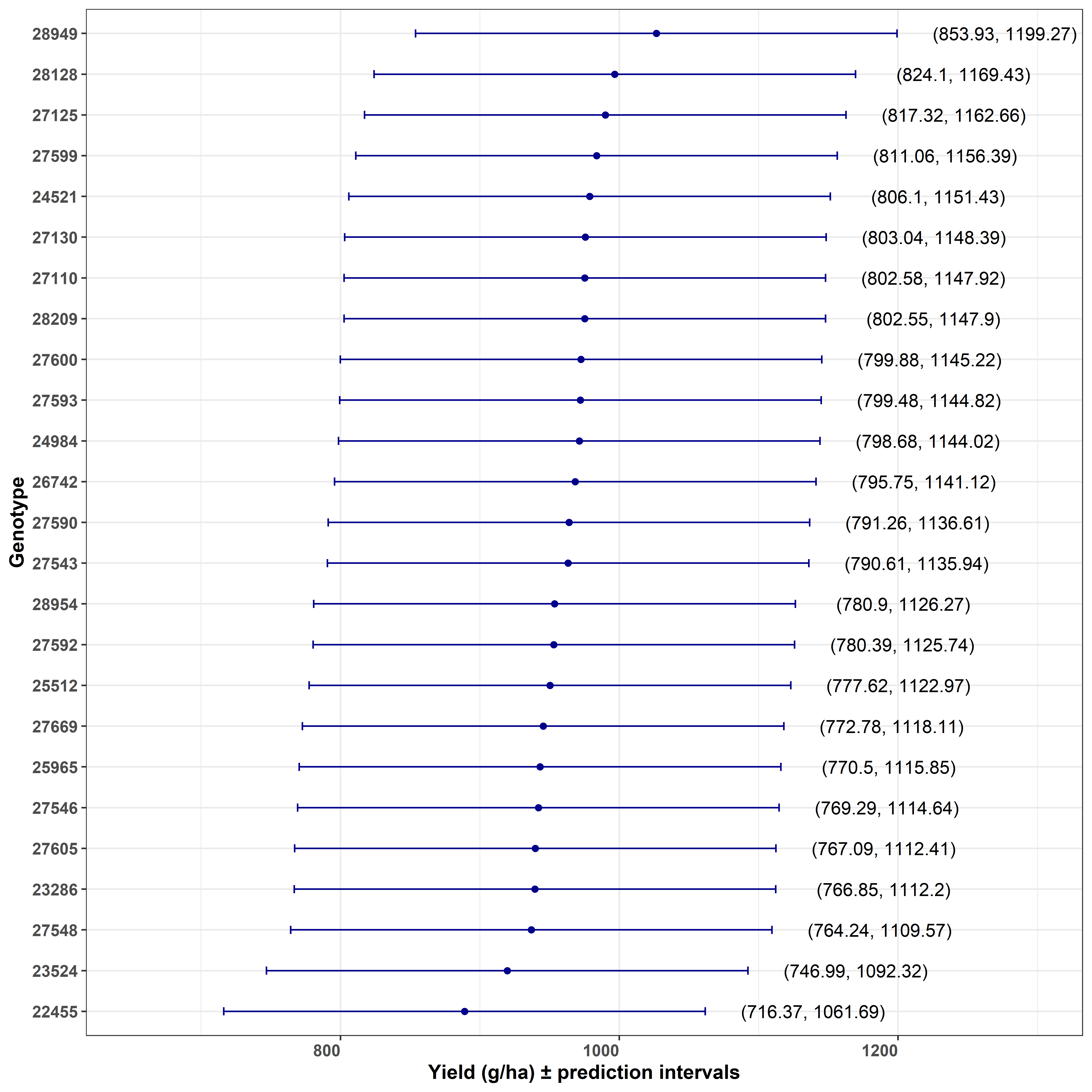

Supplement: Supplementary file 1 — Supplementary file1 (ZIP 1921kb) [file 122_2021_3786_MOESM1_ESM.zip › 122_2021_3786_MOESM1_ESM/Prediction_Intervals_Plot_R.png]
